# Axitinib in patients with advanced/metastatic soft tissue sarcoma (Axi-STS): an open-label, multicentre, phase II trial in four histological strata

**DOI:** 10.1038/s41416-023-02416-6

**Published:** 2023-09-08

**Authors:** Penella J. Woll, Piers Gaunt, Charlotte Gaskell, Robin Young, Charlotte Benson, Ian R. Judson, Beatrice M. Seddon, Maria Marples, Nasim Ali, Sandra J. Strauss, Alexander Lee, Ana Hughes, Baljit Kaur, David Hughes, Lucinda Billingham

**Affiliations:** 1 University of Sheffield, Sheffield UK and Sheffield Teaching Hospitals NHS Foundation Trust, Sheffield, S10 2JF UK; 2 Cancer Research UK Clinical Trials Unit, University of Birmingham, Birmingham, UK; 3 Sarcoma Unit, Royal Marsden Hospital, London, UK; 4 Department of Oncology, University College London Hospital, London, UK; 5 St James’s University Hospital, Leeds, UK; 6 The Clatterbridge Cancer Centre NHS Foundation Trust, Wirral, UK; 7 The Christie Hospital, Manchester, UK

**Keywords:** Sarcoma, Phase II trials

## Abstract

**Background:**

Axitinib is an oral vascular endothelial growth factor receptor inhibitor with anti-tumour activity in renal, thyroid, and pancreatic cancer.

**Methods:**

Axi-STS was a pathologically-stratified, non-randomised, open-label, multi-centre, phase II trial of continuous axitinib treatment in patients ≥16 years, performance status ≤2, with pathologically-confirmed advanced/metastatic soft tissue sarcoma (STS). Patients were recruited within four tumour strata, each analysed separately: angiosarcoma, leiomyosarcoma, synovial sarcoma, or other eligible STSs. The primary outcome was progression-free survival at 12 weeks (PFS12). A Simon’s two-stage design with activity defined as PFS12 rate of 40% determined a sample size of 33 patients per strata.

**Results:**

Between 31-August-2010 and 29-January-2016, 145 patients were recruited: 38 angiosarcoma, 37 leiomyosarcoma, 36 synovial sarcoma, and 34 other subtypes. PFS12 rate for each stratum analysed was 42% (95% lower confidence interval (LCI); 29), 45% (95% LCI; 32), 57% (95% LCI; 42), and 33% (95% LCI; 21), respectively. There were 74 serious adverse events including two treatment-related deaths of pulmonary haemorrhage and gastrointestinal bleeding. Fatigue and hypertension were the most common grade 3 adverse events.

**Conclusions:**

Axitinib showed clinical activity in all STS strata investigated. The adverse event profile was acceptable, supporting further investigation in phase III trials.

**Clinical Trial Registration:**

ISRCTN 60791336

## Introduction

Soft tissue sarcomas are rare mesenchymal tumours that can occur at any site [1]. Although accounting for less than 1% of malignant tumours, they are divided into more than 150 clinically and biologically distinct subtypes [1]. For the majority of patients with locally advanced or metastatic soft tissue sarcoma, doxorubicin-based chemotherapy remains the standard first-line treatment. However, progress is being made in identifying biologically-targeted treatments for different subtypes.

Angiogenesis is a critical process in the development of many malignancies, including soft tissue sarcomas. Circulating levels of the angiogenic factors vascular endothelial growth factor (VEGF) and basic fibroblast growth factor (bFGF) are raised in patients with soft tissue sarcoma and correlate to disease extent and risk of recurrence [2, 3]. In particular, angiosarcomas are tumours of endothelial cell differentiation [4]. They express vascular markers, including CD34, CD31, von Willebrand factor and Ulex europaeus agglutinin 1, with up-regulation of genes related to angiogenesis, including VEGF and its receptors VEGFR-1 (FLT1), VEGFR-2 (KDR), angiopoietin, PTPRB and Notch-1 [4, 5]. The PALETTE randomised phase III trial demonstrated that VEGFR inhibition significantly prolonged progression-free survival rates in patients with advanced soft tissue sarcomas [6]. However, when this study was initiated, the effects of VEGFR inhibition had not been tested in angiosarcoma.

Axitinib is an oral tyrosine kinase inhibitor targeting VEGFR-1, VEGFR-2, VEGFR-3, platelet-derived growth factor receptor (PDGFR)-β and KIT, blocking autophosphorylation and inhibiting endothelial proliferation and survival [7]. Pre-clinical studies demonstrated inhibition of tumour angiogenesis [8] and, at the time of this trial’s inception, anti-tumour activity had only been observed in clinical trials of renal cell cancer [9, 10], thyroid cancer [11], and pancreas cancer (in combination with gemcitabine) [12] with tolerable side effects [13]. We, therefore, designed a phase II clinical trial, Axi-STS, to evaluate the therapeutic activity, safety and tolerability of axitinib in four strata of patients with advanced/metastatic angiosarcoma, leiomyosarcoma, synovial sarcoma and other soft tissue sarcomas (based on our interest in angiosarcoma and the activity of pazopanib in non-adipocytic sarcomas [14]) who had relapsed after standard chemotherapy. This paper reports the final results from this trial.

## Methods

### Study design and participants

Axi-STS was a pathologically-stratified, non-randomised, open-label, multi-centre, phase II clinical trial of oral axitinib in patients with advanced soft tissue sarcoma. Patients were recruited to one of four separate pathological strata according to the World Health Organisation 2002 classification for soft tissue tumours [15], and each analysed separately: (i) angiosarcoma (and other malignant vascular tumours including epithelioid haemangioendothelioma and Kaposi sarcoma); (ii) leiomyosarcoma (both uterine and extra-uterine including skin); (iii) synovial sarcoma; and (iv) other intermediate/high-grade sarcoma subtypes excluding osteosarcoma, Ewing, chondrosarcoma, gastrointestinal stromal tumour, dermatofibrosarcoma protuberans, malignant mesothelioma and mixed mesodermal tumours of the uterus. A retrospective central pathology review of tumour histology was performed.

Eligible patients were aged ≥16 years, WHO performance status ≤2, with pathologically confirmed locally advanced or metastatic soft tissue sarcoma, incurable by surgery or radiotherapy, and had received no more than two prior lines of chemotherapy for advanced disease. Patients had measurable disease according to RECIST 1.1 criteria [16], and objective evidence of disease progression in the preceding six months. Adequate renal, hepatic, cardiac, and bone marrow function was required, and blood pressure had to be controlled (≤140/90 mmHg); anticoagulant therapy with low molecular weight heparin was permitted, but antiplatelet medication including aspirin >325 mg/day was not. Concurrent medications known to induce CYP3A4 or CYP1A2, or potent inhibitors of CYP3A4 were not permitted. Previous exposure to angiogenesis inhibitors was not an exclusion.

Following the reporting of two suspected unexpected serious adverse reactions (SUSARs), patients with cavitating lung metastases, or any metastases abutting or invading a major pulmonary blood vessel, were also deemed ineligible; and patients were excluded if they had a history of haemoptysis. Furthermore, exclusion due to history of bleeding was extended from the previous three to 12 months, and with chest x-rays added at four and eight weeks into the trial patient pathway. The full eligibility criteria are detailed in the trial protocol (supplementary appendix 1).

Procedures

Axitinib (Inlyta, Pfizer) was taken orally as 5 mg tablets, twice daily continuously in 28-day cycles until disease progression, death, unacceptable toxicity, or withdrawal of patient consent. One dose reduction to 3 mg twice daily was permitted.

The pre-treatment evaluation included medical history, physical examination, full blood count, clinical biochemistry, urinary protein, chest x-ray, 12 lead-ECG and cardiac ECHO/MUGA. Disease assessment via CT or MRI scans and photos (where indicated) were performed within four weeks prior to starting trial treatment, and then 12-weekly until disease progression. Treatment responses were assessed by RECIST 1.1 [16]., which were centrally reviewed by the trial radiologist. Chest x-rays were repeated at weeks four, eight, 12 and then 12-weekly thereafter until progression unless CT/MRI was performed at the same time point and included the whole thorax. Between study visits, patients measured their own blood pressure at home twice daily prior to taking each dose of axitinib using provided automatic sphygmomanometers.

Adverse events (AE) according to NCI-CTCAE v4.03 [17] were recorded weekly in the first cycle and four-weekly thereafter. Adverse events were managed with predefined dose and schedule modifications; in the event of any grade ≥3 AE, axitinib treatment was paused until the AE had recovered to grade ≤1, when axitinib could be reintroduced at a lower dose of 3 mg twice daily. Treatment interruption was permitted for a maximum of two weeks. If the AE had not improved sufficiently within this time, the patient’s trial treatment was discontinued.

After disease progression, patients were followed up every three months for survival.

Outcomes

The primary outcome measure was progression free-survival at 12 weeks from trial entry (PFS12), in patients who received at least one cycle of treatment; patients who were alive with stable or responding disease at this time were defined as success. Secondary outcome measures included tumour response (i.e., complete or partial response as per RECIST 1.1 [16]) at 12 weeks, best percentage change in size of target lesions, progression-free survival time (PFS), progression-free interval (PFI), overall survival time (OS), changes in WHO performance status from baseline to the end of treatment cycle 3, and toxicity defined as the occurrence of at least one grade 3 or 4 AE or treatment-related serious adverse event (SAE) in patients who started treatment. Analyses of duration of response (time from response to progression, death, or date last seen), as well as baseline performance status on clinical response and modified Glasgow Prognostic Score (GPS), are reported as *post-hoc* analyses in response to reviewer comments.

Circulating biomarker analysis

Serum samples for biomarker analysis were collected pre-treatment, and at the start of each cycle until treatment discontinuation. Peripheral blood was collected in 8.5 ml BD no additive Vacutainers, kept at room temperature for 30 min to ensure complete clotting, and then centrifuged at 2000 *g* for 20 min at room temperature. The resulting supernatant was aliquoted and stored at −80 ^o^C until analysis. Previous studies to evaluate circulating biomarkers have typically investigated pre-determined markers. To cast a wider net, we employed an unselected proteomic approach using Isobaric Tags for Relative and Absolute Quantification (iTRAQ). A training set of four groups of serum samples was established: samples collected from four leiomyosarcoma patients classified as good responders (PFS on axitinib ≥4 months), pre-treatment and after four weeks of therapy, and parallel samples from four age and sex-matched leiomyosarcoma patients classified as poor responders (PFS on axitinib <4 months). The serum samples in the four groups were pooled and depleted of high-abundant proteins using Pierce Top 12 Abundant Protein Depletion Spin Columns according to the manufacturer’s instructions. The four groups were compared using a quantitative mass spectrometry-based proteomic workflow to determine differences in protein level between sample groups, and the relative expressions of identified proteins compared between groups, to identify candidate biomarkers [18]. Commercial enzyme-linked immunosorbent assays (ELISA) from Cusabio were used according to manufacturer instructions to subsequently quantify expression levels of circulating actin, cytoplasmic 2 (ACTG1) pre-treatment and after four weeks of axitinib, and C-reactive protein (CRP) pre-treatment, in the wider patient population.

Statistical analysis

The trial used a Simon’s two-stage phase II minimax design, with each pathological stratum analysed separately. Consistent with EORTC Soft Tissue and Bone Sarcoma Group guidance [19], a 12-week PFS rate of ≥40% (P1) indicated an active drug worthy of further study, while ≤20% (P0) suggested drug inactivity. The trial was designed with one-sided type I and type II error rates of 0.05 and 0.20, respectively. Based on these criteria, if ≥11 successes were seen in 33 eligible treated patients within a pathological stratum, the drug was deemed active. To allow for ineligible or untreated patients up to five additional patients were allowed within each stratum, up to a maximum of 38 per stratum and 152 for the trial.

An independent data monitoring committee (DMC) reviewed interim data annually to ensure patient safety. A planned interim analysis was scheduled in each stratum when 18 patients had completed a minimum of 12 weeks follow-up. If fewer than five successes were observed, recruitment to the stratum would be closed; if five or more successes were observed, the stratum would continue to recruit.

The final analysis of the primary outcome was conducted using a modified intention-to-treat (ITT) approach and took place after all patients within a stratum had been followed for a minimum of 12 weeks and is reported as PFS12 rate with a one-sided 95% lower confidence interval (LCI) in accordance with the type I error rate used in the design. The tumour response rate is reported as the proportion of patients who experience complete or partial response with 95% confidence intervals (CI). PFS, PFI, and OS were analysed using the Kaplan-Meier method of estimation, with median survival and survival rates at one year reported alongside 95% CI. Baseline performance status is reported against the status recorded at the end of cycle 3. Toxicity rates are reported with 95% CI.

For the analysis of PFS12 and tumour response at 12 weeks, evaluable patients were defined as those patients who received at least one cycle of treatment. For all other outcomes, evaluable patients were defined as those who received at least one dose of treatment. Both populations included ineligible patients who received sufficient treatment to be considered evaluable, if the reason for ineligibility was not deemed to influence the outcome being analysed. For PFI, patients who died of disease without any recorded disease progression have been defined as having progressed at the date of death, whilst those who died of other causes were censored at their date of death. For PFS and PFI, patients alive and progression-free at the time of analysis were censored at their date last known to be in this state. Similarly, for OS, patients alive at the time of analysis were censored at the date last seen alive.

The best percentage change from baseline in the total sum of diameters of target lesions is presented incorporating the best response (at any timepoint) of each patient using waterfall plots; the total sum of diameters (measured in mm) at baseline was calculated and compared to each subsequent disease evaluation visit.

Analyses were performed using Stata 17.0.

The trial was prospectively registered: ISRCTN 60791336.

## Results

**Fig. 1:**
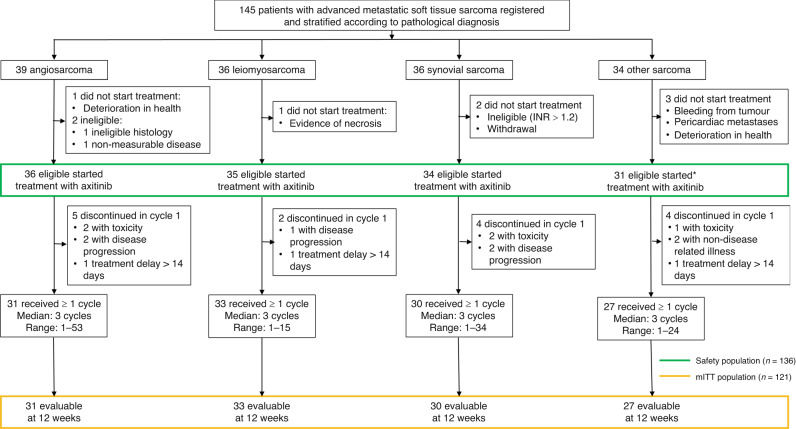
Axi-STS trial profile. The diagram shows the flow of patients through the four separate sarcoma strata of the Axi-STS trial. *Although the cohort target was 33 eligible patients and 34 patients were recruited to the other sarcoma stratum, following closure to recruitment and data cleaning it became apparent that three patients were not able to start treatment reducing the number to 31. mITT, modified intension-to-treat.

Between 31-August-2010 and 29-January-2016, 145 sarcoma patients were recruited through 13 UK centres; 39 angiosarcoma, 36 leiomyosarcoma, 36 synovial sarcoma, and 34 other subtypes (Fig. 1). Patient characteristics and disease history at baseline are described in Table 1. The median age of all patients in the trial was 56 years (range 20, 82), with 63 males (43%) and 134 WHO performance status ≤1 (92%). This was broadly similar across the four soft tissue sarcoma strata although the median age of synovial sarcoma patients was lower (44 years; range 20, 73), and more leiomyosarcoma patients were female (26/36; 72%). The majority of patients recruited had Trojani grade 2 or 3 sarcomas (111/145; 77%) (Table 1). The pathology of the soft tissue sarcomas included in the “other” stratum were six solitary fibrous tumour, four pleomorphic soft tissue sarcoma, four spindle cell soft tissue sarcoma, four alveolar soft part sarcoma, four myxoid liposarcoma, two endometrial stromal sarcoma, with one of each of the following: undifferentiated pleomorphic sarcoma, sclerosing epithelioid fibrosarcoma, pleomorphic spindle cell sarcoma, myxoid and round cell liposarcoma, malignant peripheral nerve sheath tumour, liposarcoma, low-grade fibromyxoid sarcoma, malignant phyllodes tumour, desmoplastic small round cell tumour, and dedifferentiated liposarcoma. Among the 39 angiosarcomas there was one Kaposi sarcoma and five haemangioendotheliomas.

**Table 1:** Patient characteristics

	Angiosarcoma	Leiomyosarcoma	Synovial Sarcoma	Other Sarcoma	Total
	*N* = 39	*N* = 36	*N* = 36	*N* = 34	*N* = 145
**Patient baseline characteristics,** ***n*** **(%)**					
**Age (years)**					
Median	64	59	44	50	56
Interquartile range	55, 72	51, 63	32, 59	38, 65	42, 65
Range	27, 82	29, 79	20, 73	21, 80	20, 82
≥65	18 (46)	7 (19)	5 (14)	9 (26)	39 (27)
**Sex**					
Male	14 (36)	10 (28)	18 (50)	21 (62)	63 (43)
Female	25 (64)	26 (72)	18 (50)	13 (38)	82 (57)
**WHO Performance Status**					
0	18 (46)	10 (28)	16 (44)	7 (21)	51 (35)
1	18 (46)	22 (61)	18 (50.0)	25 (73)	83 (57)
2	3 (8)	3 (8)	2 (6)	2 (6)	10 (7)
Unknown	0	1 (3)	0	0	1 (1)
**Trojani Tumour Grade**					
1	3 (8)	2 (6)	0	2 (6)	7 (5)
2	9 (23)	12 (33)	11 (31)	7 (21)	39 (27)
3	20 (51)	16 (44)	20 (56)	16 (47)	72 (50)
Not supplied	0	0	0	1 (3)	1 (1)
Unknown	7 (18)	6 (17)	5 (14)	8 (24)	26 (18)
**Primary Tumour Location,** ***n*** **(%)**					
Liver	2 (5)	1 (3)	0	1 (3)	4 (3)
Lymph	1 (3)	0	0	0	1 (1)
Lung	2 (5)	0	4 (11)	4 (12)	10 (7)
Bone	1 (3)	0	0	2 (6)	3 (2)
Other, soft tissue	1 (3)	1 (3)	1 (3)	0	3 (2)
Upper limb	1 (3)	1 (3)	2 (6)	1 (3)	5 (3)
Lower limb	5 (13)	9 (25)	16 (44)	9 (26)	39 (27)
Shoulder girdle	0	0	2 (6)	1 (3)	3 (2)
Pelvic girdle	0	2 (6)	1 (3)	5 (15)	8 (6)
Breast	9 (23)	0	0	1 (3)	10 (7)
Head and neck	7 (18)	0	1 (3)	0	8 (6)
Other, intra-abdominal	2 (5)	8 (22)	5 (14)	4 (12)	19 (13)
Other, trunk	4 (10)	0	3 (8)	3 (9)	10 (7)
Uterus	0	12 (33)	0	2 (6)	14 (10)
Other	4 (10)	2 (6)	1 (3)	1 (3)	8 (6)
**Systolic Blood Pressure (mmHg)**					
Mean	127.3	124.5	120.4	124.2	124.2
Standard deviation	17.8	12.6	16.9	13.6	15.5
Range	96.0, 176.0	104.0, 154.0	80.0, 184.0	95.0, 148.0	80.0, 184.0

A table of the patient baseline characteristics within the Axi-STS trial.

At the time of data lock (30-Jun-2022) there were 122 reported deaths across the four strata. Median follow-up time was 73.5 months with 91% of patients followed for more than 36 months.

Following registration into the trial and prior to treatment, one angiosarcoma patient was found to be ineligible due to haemoptysis, one leiomyosarcoma patient had evidence of tumour necrosis with a high-risk of bleeding, one synovial sarcoma patient was found to have an INR > 1.2 with a second withdrawing as they were reluctant to take the treatment, and within the other sarcoma subtype stratum one patient developed bleeding from their tumour, a second was found to have peri-cardiac metastases and a third was deemed too unwell by their physician to start treatment (Fig. 1). Of the 138 patients (95%) who started cycle 1 of protocol treatment, 15 discontinued treatment within cycle 1 due to rapid disease progression (*N* = 5), unacceptable toxicity (*N* = 5), a treatment delay of more than 14 days (*N* = 3) or a non-disease related illness (*N* = 2) and were excluded from response assessment. In addition, two angiosarcoma patients were subsequently found not to be evaluable due to a local review of the patient’s histology including their molecular histology (not originally available at diagnosis), which subsequently reclassified their disease as extraskeletal myxoid chondrosarcoma; lymph node disease was smaller than permitted as per RECIST v1.1 in a second patient. The remaining 121 patients were included in the modified ITT analysis.

The time to starting treatment, the number of cycles of axitinib received, and the number of dose reductions and interruptions observed across all four strata were similar, although patients with angiosarcoma on average received more cycles of treatment (supplementary appendix 2). Disease progression was reported as a contributing factor for treatment discontinuation in 98 patients (71%), with toxicity a contributing factor for 19 patients (14%); 73 patients (53%) went on to receive further anti-tumour treatments once axitinib had ceased (supplementary appendix 2).

The interim analysis concluded that recruitment within all four strata could continue as all exceeded the minimum requirement for PFS12 of five “successes”. The proportion meeting the definition of success for the primary endpoint were: 9/18 for angiosarcoma, 7/18 for leiomyosarcoma, 10/18 for synovial sarcoma, and 6/18 for the other sarcoma stratum. At the final analysis, 54 patients of the 121 evaluable patients achieved PFS12 (45%, 95% LCI: 37) (Table 2). Three of the strata achieved the minimum requirement for PFS12 of 11 successes to be deemed active: 13/31 (42%; 95% LCI: 29) for angiosarcoma, 15/33 (45%; 95% LCI: 32) for leiomyosarcoma, 17/30 (57%; 95% LCI: 42) for synovial sarcoma. The other sarcoma stratum did not reach the required 33 evaluable patient target (see Fig. 1 footnote): 9 successes were observed in the 27 evaluable patients giving PFS12 rate of 33% (95% LCI: 21%). This was above the 20% inactivity threshold specified in the design and, therefore, axitinib was also deemed active in this fourth stratum.

**Table 2:** Axi-STS trial outcomes

	Angiosarcoma	Leiomyosarcoma	Synovial Sarcoma	Other Sarcoma	Total
12-week PFS [% (95% LCI)]	42 (29)	45 (32)	57 (42)	33 (21)	45 (37)
Proportion	13/31	15/33	17/30	9/27	54/121
Tumour response rate at 12 weeks [% (95% CI)]	6 (2, 21)	0 (0, 10)	10 (3, 26)	4 (1, 18)	5 (2, 10)
Proportion	2/31	0/33	3/30	1/27^a^	6/121
Median PFS [months (95% CI)]	3.0 (2.4, 6.8)	2.8 (2.6, 5.1)	3.1 (2.2, 5.4)	2.8 (2.5, 3.8)	2.9 (2.6, 3.8)
12-month PFS [% (95% CI)]	19 (9, 34)	6 (1, 17)	12 (4, 25)	10 (2, 23)	12 (7, 18)
Median PFI [months (95% CI)]	3.0 (2.4, 6.8)	2.8 (2.6, 5.1)	3.2 (2.4, 5.4)	2.8 (2.5, 3.8)	2.9 (2.6, 3.8)
12-month PFI [% (95% CI)]	22 (10, 36)	6 (1, 17)	12 (4, 26)	10 (2, 23)	12 (7, 19)
Median OS [months (95% CI)]	8.6 (5.3, 17.8)	11.4 (9.2, 12.6)	9.7 (7.5, 17.6)	9.9 (6.7, 14.1)	9.9 (9.1, 12.0)
12-month OS [% (95% CI)]	40 (24, 56)	40 (24, 56)	47 (30, 63)	34 (18, 50)	40 (32, 49)
Toxicity^b^ rate [% (95% CI)]	72 (54, 84)	60 (44, 74)	68 (51, 81)	65 (47, 79)	66 (58, 74)
Proportion	26/36	21/35	23/34	20/31	90/136

The primary outcome 12-week progression-free survival, and secondary outcomes 12-week tumour response rate, median and 12-month progression-free survival time, median and 12-month progression-free interval, median and 12-month overall survival time, and toxicity rate.

*CI* confidence interval; *LCI* lower confidence interval; *PFI* progression-free interval rate; *PFS* progression-free survival rate; *OS* overall survival rate.

^a^The sarcoma type for the patient in this group that responded was endometrial stromal sarcoma.

^b^Toxicity was defined as the occurrence of at least one grade 3 or 4 adverse event or treatment-related serious adverse event.

**Fig. 2:**
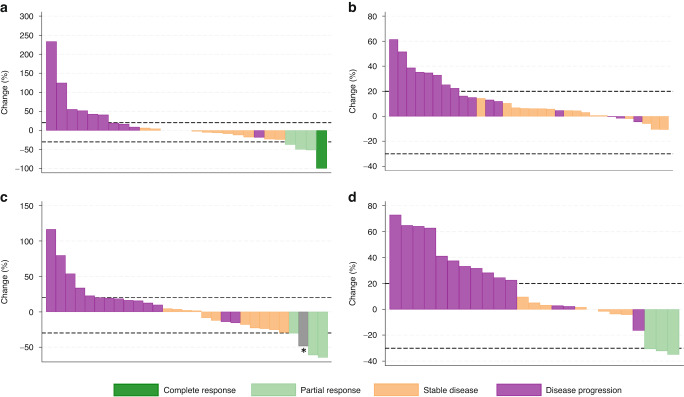
Best response of target lesions to axitinib for patients within each sarcoma stratum. At baseline the size of each target lesion (diameter in mm) at any timepoint was measured and then at each disease evaluation visit the size of each target lesion was re-measured, with the percentage change calculated. For patients whose total sum of diameters decreased the waterfall plots present the largest reported decrease in size alongside the associated overall response. For patients whose total sum of diameters increased the waterfall plot presents the smallest increase alongside the associated overall response. Panel **a** shows the angiosarcoma, **b** the leiomyosarcoma, **c** the synovial sarcoma, and **d** the other sarcoma stratum. As defined by RECIST 1.1, partial response (20%) and progression (−30%) targets are indicated by the dotted horizontal lines. N.B. The complete response recorded for one angiosarcoma patient occurred during treatment but after the initial 12 weeks. * This synovial sarcoma patient discontinued axitinib due to disease progression, then went on to receive radiotherapy and achieve a partial response. This radiological response was five months after they had discontinued axitinib. As such they have been classified differently to those who responded directly to axitinib.

In total, as assessed by RECIST v1.1, six patients achieved objective tumour responses (all partial) at 12 weeks (Table 2); the histology of these responders included three synovial sarcomas, two angiosarcomas and one endometrial stromal sarcoma. Subsequent to this time point, one patient with an angiosarcoma of the right atrium who achieved a partial response at 12 weeks, went on to achieve a complete radiological response at their week 52 scan. Their complete response was maintained for 19 months until disease progression and treatment discontinuation. Four additional patients achieved a partial response during treatment after 12 weeks, including two angiosarcomas, one alveolar soft-part sarcoma and one malignant peripheral nerve sheath tumour. For the 10 patients who responded during treatment, the median duration of response was 5.8 months (95% CI: 1.0–22.7). The best percentage change from baseline (at any timepoint) in the total sum of diameters of target lesions during the trial is shown in Fig. 2. In addition, the presence of any new lesions is shown in supplementary appendix 3.

A *post-hoc* analysis of the baseline performance status of those patients who responded revealed no patients had a baseline performance status of 2 and there were more patients who had baseline performance status of 0 compared to those who were 1 (6/10 compared to 4/10, respectively). However, the numbers are too small to draw any definitive conclusions.

A *post-hoc* analysis of the angiosarcoma patients suggested different responses to axitinib depending upon the primary tumour site. The proportion of evaluable patients with head and neck angiosarcoma without progression at 12 weeks was 4/7 compared to patients with angiosarcoma of the breast 2/9, or for other primary disease sites 5/17. However, we note that patient numbers in these subgroups are small.

**Fig. 3:**
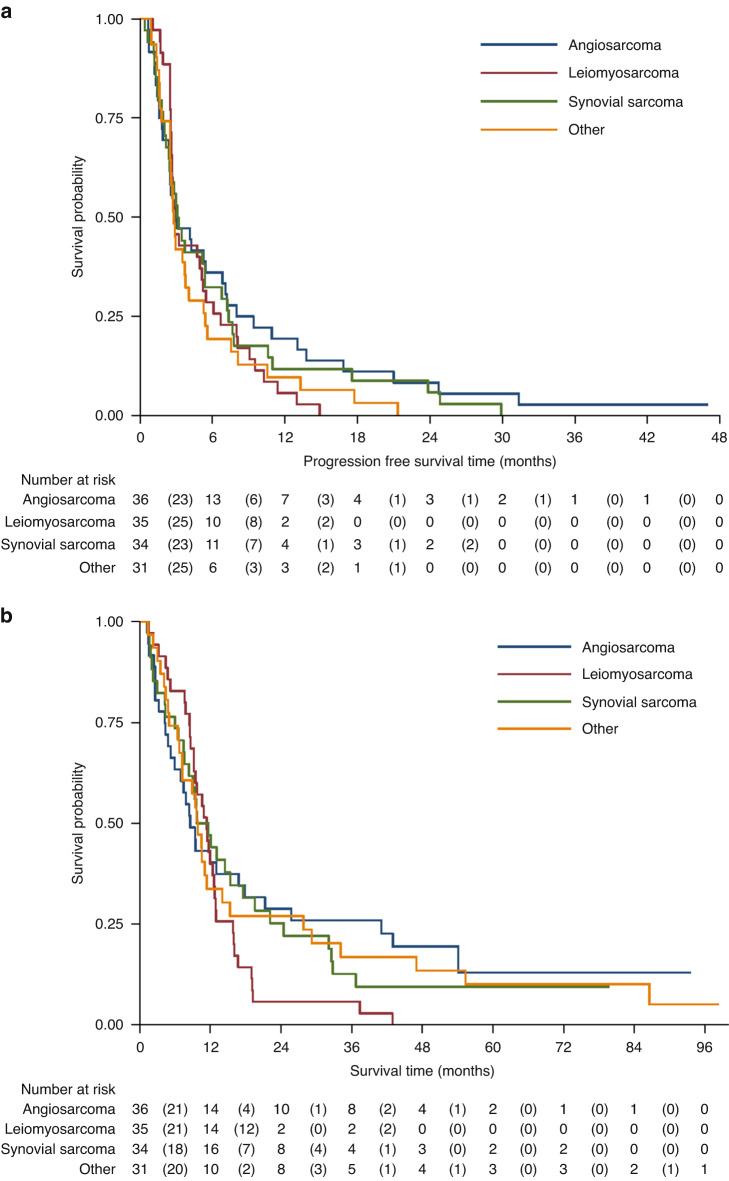
Progression-free and overall survival time of all sarcoma patients. Kaplan–Meier analyses of secondary outcome measures. Panel **a** shows disease-free survival defined as the time from trial entry to the date of first observed disease progression or date of death from any cause. Panel **b** shows overall survival defined as the time from trial entry to death from any cause. Patients were censored at date last seen. CI = Confidence Interval.

The median PFS of all sarcoma patients was 2.9 months (95%CI: 2.6, 3.8) consisting of 122 disease progressions and 13 deaths (Fig. 3a). The median and 12-month PFS for all strata are presented in Table 2. The median PFI of all sarcoma patients was also 2.9 months (95%CI: 2.6, 3.8) consisting of 122 disease progressions and 11 disease-related deaths (Table 2). Median OS for all sarcoma groups was 9.9 months (95%CI: 9.1, 12.0) consisting of 122 deaths (Fig. 3b). The median and 12-month OS for all strata are presented in Table 2.

Irrespective of performance status at baseline, the performance status of the majority of patients remained unchanged by cycle 3 of treatment, the 12-week disease evaluation time point (63/136; 46%) (supplementary appendix 4; table S4a). Nineteen patients demonstrated a deterioration in performance status by one point, one patient by two points, and seven patients demonstrated improvements in their performance status by one point. The total changes and those within each stratum are presented in figures S4a–S4e in supplementary appendix 4.

All 136 patients who started axitinib experienced at least one AE over the course of the trial. A total of 5522 AEs were reported, 210 of which were grade ≥3 or unknown: 74 within the angiosarcoma stratum, 59 within the leiomyosarcoma, 33 within the synovial sarcoma, and 44 within the other sarcoma subtype stratum (supplementary appendix 5; table S4a). Only three grade 4 events were reported during the trial: one episode of grade 4 hypercalcaemia was reported in one leiomyosarcoma patient within cycle 1 of treatment who continued treatment and had stable disease at week 12, and two patients with synovial sarcoma who had grade 4 events of dyspnoea and lung infection, respectively. Twenty-five adverse events were reported that affected at least 10% of patients across all four strata (supplementary appendix 5; table S5b); the most common were fatigue (affecting 114/136 patients−21 were grade 3) and hypertension (affecting 94/136 patients–23 were grade 3).

There were 74 SAEs reported during the trial within 56 patients: 20 (27%) were reported in 17 angiosarcoma patients, 14 (19%) in nine leiomyosarcoma patients, 26 (35%) within 20 synovial sarcoma patients, and 14 (19%) within 10 patients with other soft tissue sarcomas. Of the 35 treatment-related SAEs, four were fatal: one in the angiosarcoma stratum, two in the leiomyosarcoma stratum, and one in the other sarcoma stratum (Table 3).

**Table 3:** Trial treatment-related serious adverse events

Stratum	Admitting Event	Other Event	Reason	Grade
Angiosarcoma	Pulmonary embolus		Hospitalisation	3
	Pneumothorax	Dyspnoea	Hospitalisation	3
	Pneumothorax		Hospitalisation	3
	Disease progression^a^		Hospitalisation; Death	5
	Flank pain	Constipation; Urinary tract infection	Hospitalisation	3
	Skin disorder bleeding from metastatic tumour site	Haemoptysis	Hospitalisation	3
	Duodenal perforation	Vomiting; Fever; Anaemia	Hospitalisation	2
	Abdominal pain	Cholecystitis; Constipation	Hospitalisation	3
	Abdominal pain		Hospitalisation	3
	Fatigue	Fever; Confusion	Hospitalisation	3
	Hypertension	Headache	Hospitalisation	3
Leiomyosarcoma	Dehydration	Pyrexia	Hospitalisation	2
	Haemoptysis		Other	1
	Disease progression^a^		Hospitalisation; Death	5
	Atrial fibrillation		Hospitalisation	4
	Gastrointestinal bleed	Atrial fibrillation	Death	5
Synovial sarcoma	Cerebral metastasis with haemorrhage.		Hospitalisation	3
	Lower respiratory tract infection		Hospitalisation	2
	Respiratory Failure	Pneumonia	Life Threatening; Hospitalisation; Disability	4
	Pneumothorax		Hospitalisation	2
	Anorectal Infection		Hospitalisation	3
	Hypocalcaemia		Hospitalisation	3
	Pneumothorax	Venous thrombosis	Hospitalisation	2
	Chest infection	Pneumothorax	Hospitalisation	2
	Dyspnoea		Hospitalisation	2
	Herpes Zoster		Other	2
	Bilateral pneumothoraces		Hospitalisation	3
	Lower gastrointestinal haemorrhage	Pelvic pain	Hospitalisation; Life Threatening	3
	Dyspnoea	Pleural haemorrhage; Pleural effusion	Hospitalisation; Disability	3
	Left retinal vein occlusion		Other	3
Other sarcoma subtype	Pneumothorax		Hospitalisation	3
	Haemoptysis	Chest pain	Death	5
	Pain central chest		Hospitalisation	2
	Wound infection/Abscess		Hospitalisation	3
	Leaking right renal artery	Tumoural pulmonary emboli	Hospitalisation	3

A list of those serious adverse events that were deemed to be related to trial treatment during Axi-STS.

^a^In both these cases the patients died of disease progression. The Principal Investigator assigned causality of their general decline, which led to death, to be at least possibly related to trial medication. Therefore, both were retained as treatment-related SAEs.

Dashed horizontal lines indicate those events that were experienced by the same patient.

Analysis of the serum sample proteomes showed clustering of the pre-treatment samples from the good responders away from the other sample groups (supplementary appendix 6, figure S6a). Further proteome analysis to identify candidate biomarkers, therefore, focused on comparisons between the pre-treatment samples of the good responders versus poor responders. ACTG1, a cell junction assembly protein involved in the ephrin and VEGF receptor signalling pathways, was selected as a putative candidate biomarker for further analysis, as it showed a high fold change relative to other candidate biomarkers and appeared biologically relevant to the mechanism of action of axitinib (supplementary appendix 6, table S6a). ACTG1 levels in the wider patient population, as measured by ELISA, did not differ when pre-treatment and four weeks post-treatment were compared, or by tumour grade or tumour strata; there was also no difference in PFS by pre-treatment ACTG1 expression (supplementary appendix 6, figure S6b). Many of the candidate biomarkers were noted to be acute phase response proteins. The modified Glasgow Prognostic Score (mGPS) uses serum albumin and CRP (two well-establish acute phase proteins and markers of the systemic inflammatory response) to stratify patients into three groups [20]. When the mGPS was calculated for each patient, patients with mGPS 1 or 2 had a significantly worse PFS than patients with mGPS 0 (GPS 0 vs 1 or 2, median PFS 5.6 months (95% CI; 3.7, 8.0) vs 2.5 months (95% CI; 1.9, 2.7); log-rank *p* < 0.001); supplementary appendix 6, figure S6c. A *post-hoc* analysis to explore if there was any relationship between baseline performance status and mGPS revealed that there was a higher proportion of patients with baseline performance status 1 or 2 in the mGPS 1 or 2 group (49/64; 77%) compared to the mGPS 0 group (21/49; 43%).

## Discussion

Until recently, all soft tissue sarcomas were treated in the same way, but in the last decade, progress has been made in identifying clinical, histological and molecular features to guide management [21]. New agents are typically evaluated in phase II studies with PFS as the primary endpoint. An analysis of 13 phase II studies conducted by the EORTC Soft Tissue and Bone Sarcoma Group concluded that a 12-week PFS rate of ≥40% (P1) indicated an active drug worthwhile for further study, while ≤20% (P0) suggested drug inactivity [19]. In a subsequent phase II study of pazopanib, these criteria were used to determine that pazopanib had activity in leiomyosarcoma, synovial sarcoma and other soft tissue sarcomas but not liposarcoma [14]. The activity of pazopanib was then confirmed in a phase III study in non-adipocytic soft tissue sarcomas [6], vindicating this screening approach. Using this methodology, we have demonstrated the activity of axitinib (P1 > 40%) in angiosarcoma, leiomyosarcoma, synovial sarcoma and other soft tissue sarcoma strata. In total, 10 objective tumour responses were observed during treatment: four in angiosarcomas, three in synovial sarcomas, and three in the other sarcoma strata (one of endometrial stromal sarcoma, one alveolar soft part sarcoma, and one malignant peripheral nerve sheath tumour). Due to differences in trial design and the heterogeneity of the study patient populations, it is difficult to directly compare our results with other studies in advanced soft tissue sarcoma of multi-tyrosine kinase inhibitors such as pazopanib [6] and regorafenib [22]; however together they support a drug class effect.

Angiosarcomas are rare malignant endothelial cell tumours. The standard treatments for advanced disease include anthracyclines, paclitaxel and gemcitabine, but there has been particular interest in evaluating anti-angiogenic agents in this disease [23]. Axitinib (Inlyta, Pfizer) is a potent orally bioavailable tyrosine inhibitor with activity against VEGFR-1, −2 and −3, and PDGFR-β, thereby exerting an anti-angiogenic effect. This study has demonstrated that axitinib has activity in angiosarcoma but was not able to determine the mechanism for this. When this study was initiated, the effects of VEGFR inhibition had not been tested in angiosarcoma. Recently, several groups have studied pazopanib in angiosarcoma, alone or in combination with other agents [24–26]. Their findings have been broadly in line with our own for PFS and response rate.

Because angiosarcomas are rare tumours, clinical trials in this patient group are challenging. Patients often have rapidly progressive disease at relapse after chemotherapy. In addition, the geographic nature of lesions can make formal response assessment challenging. For this reason, we included photographic assessment of lesions and external radiological response review was undertaken. Here, we report a formal phase II study of axitinib in four patient strata: angiosarcoma, leiomyosarcoma, synovial sarcoma and other soft tissue sarcomas. In some sarcoma subtypes, treatment with multi-kinase inhibitors has been associated with changes in tumour density rather than lesion size. Modified response criteria have been proposed by Choi et al. [27], to capture this. In our study, an assessment of lesions was performed using the Choi criteria retrospectively on a limited subset of patients (*N* = 25). Data have not been presented but there was little change in tumour density and no evidence that the Choi criteria would be superior to RECIST in describing this data set.

Axitinib was well tolerated during Axi-STS. The AE profile has been carefully documented in previous studies, and as expected, the commonest toxicities were fatigue, hypertension, oral mucositis and nausea. However, two patients with cavitating lung metastases experienced significant haemoptysis, which was fatal in one. As described in the Methods section, we updated the original eligibility criteria so that patients were excluded if they had a history of haemoptysis and patients with cavitating lung metastases, or any metastases abutting or invading a major pulmonary blood vessel, became ineligible.

The mechanism of action of anti-angiogenic agents in the treatment of soft tissue sarcoma remains uncertain but potentially involves direct inhibition of tumour cells, as well as effects on the tumour micro-environment, including interaction with the tumour immune microenvironment. Both tumour mutational analyses and studies of circulating cytokines and angiogenic factors have failed to identify validated biomarkers to predict response to these agents [28, 29]. We took an unselected approach to screen serum samples collected from patients recruited to Axi-STS for circulating proteomic biomarkers using iTRAQ technology. Unfortunately, our subsequently selected biomarker was similarly unsuccessful, and ultimately it is most likely that soft tissue sarcoma response to multi-tyrosine kinase inhibitors will best be predicted by a signature of multiple biomarkers rather than through a single item [30]. The mGPS has previously been evaluated in patients with localised soft tissue sarcoma [31]. Here, we have shown that it is prognostic in patients with advanced disease too. Treatment decision-making in patients with soft tissue sarcoma is often complex; systemic treatment options often have significant side effects, and the benefits are often relatively modest. The mGPS is worth further evaluation in patients with advanced soft tissue sarcoma to help inform these decisions [32].

As with all trials, several limitations can be identified. Of note, the details of prior systemic treatment and whether sarcomas were metastatic or locally advanced/unresectable were not collected during Axi-STS. Neither was the presence of liver metastasis separately collected, which has recently been reported as an indicator of poor prognosis [33]. In addition, detailed subtyping of those patients recruited into the angiosarcoma, leiomyosarcoma, and synovial sarcoma strata was not consistently reported. Taken together, these have limited the subgroup analyses possible, which may have constrained some interpretations of the clinical activity in particular sarcoma subtypes. We note, however, that due to the small numbers recruited in this phase II trial, any further sub-analyses would have lacked validity.

In conclusion, the Axi-STS trial has demonstrated preliminary clinical activity of the VEGFR inhibitor axitinib across four strata of patients with advanced/metastatic soft tissue sarcomas. The drug was well tolerated and merits further investigation in a phase III trial in patients with angiosarcoma, leiomyosarcoma, synovial sarcoma, and another non-adipocytic intermediate/high-grade sarcoma.

### Supplementary information

Axi-STS Appendices

## Data Availability

Participant data and the associated supporting documentation will be available within six months after the publication of this manuscript. Details of our data request process are available on the CRCTU website. Only scientifically sound proposals from appropriately qualified research groups will be considered for data sharing. The decision to release data will be made by the CRCTU Director’s Committee, which will consider the scientific validity of the request, the qualifications and resources of the research group, the views of the Chief Investigator and the trial steering committee, consent arrangements, the practicality of anonymising the requested data and contractual obligations. A data-sharing agreement will cover the terms and conditions of the release of trial data and will include publication requirements, authorship and acknowledgements and obligations for the responsible use of data. An anonymised encrypted dataset will be transferred directly using a secure method and in accordance with the University of Birmingham’s IT guidance on the encryption of datasets.
