# Supplementary material for: Axitinib in patients with advanced/metastatic soft tissue sarcoma (Axi-STS): an open-label, multicentre, phase II trial in four histological strata
Source: Br J Cancer. 2023 Sep 8;129(9):1490–9. doi: 10.1038/s41416-023-02416-6 (PMC10628187; doi:10.1038/s41416-023-02416-6)
Supplement: Supplementary file 1 — Axi-STS Appendices [file 41416_2023_2416_MOESM1_ESM.pdf]

Supplementary appendix 1

NCRN National Cancer Research Network

Sponsor Protocol Number:	STH15195
EudraCT Number:	2008-006007-23
CRCTU Protocol Number:	SA2013
ISRCTN:	60791336

NCRI Sarcoma Clinical Studies Group

AXI-STS

A clinicopathological phase II study of axitinib in patients with advanced angiosarcoma and other soft tissue sarcomas

PROTOCOL VERSION/DATE

Version 7.0 12th April 2016

The following amendments are incorporated into this protocol

Amendment Number	Version Number	Date
Modified Substantial Amendment 1	AM01/1	01 March 2010
Substantial Amendment 3	AM03	27 September 2011
Modified Substantial Amendment 8	Modified AM08	22 May 2014
Substantial Amendment 10	AM10	01 December 2014
Substantial Amendment 11	AM11	12 April 2016

Sheffield Teaching Hospitals NHS
NHS Foundation Trust

CANCER RESEARCH UK

UNIVERSITY OF BIRMINGHAM

CLINICAL TRIAL PROTOCOL

Every care has been taken in writing this document, but corrections or amendments may be necessary. These will be circulated to the known investigators in the trial, but sites entering patients for the first time are advised to contact the trial office to confirm they have the most recent version.

Independent scientific peer review

This protocol has been submitted to independent peer review through the NCRI Sarcoma Clinical Studies Group and the Cancer Research UK, Clinical Trials Advisory and Awards Committee.

The protocol has been authorised by

Role	Name	Signature	Date
Sponsor Representative	Prof. Simon Heller		22 SEP 2011
Chief Investigator	Prof. Penella Woll		27.09.2011
Co-Investigator and Lead Biostatistician	Prof. Lucinda Billingham		22-09-2011
Co-Investigator and Histopathologist	Dr. David Hughes		19/09/11
Co-investigator and Radiologist	Dr. Christine Ingram		16/9/11

INVESTIGATOR'S AGREEMENT

Title: **Phase II trial of axitinib in sarcoma**

Protocol version number and date:

I have read and agree to the protocol as detailed in this document.
I am aware of my responsibilities as an Investigator under the EU Clinical Trials Directive (2001/20/EC), guidelines of Good Clinical Practice (GCP), the Declaration of Helsinki (October 1996), local regulations (as applicable) and the trial protocol and I agree to conduct the trial according to these guidelines and to appropriately direct and assist the staff under my control, who will be involved in the trial.

Name of Institution	
Principal Investigator's Name	
Principal Investigator's Signature	
Date	

The Principal Investigator should sign and submit the original copy of this page to the trial office.

PROTOCOL SYNOPSIS

Title

A clinicopathological phase II study of axitinib in patients with advanced angiosarcoma and other soft tissue sarcomas.

Design

An open-label, multicentre, stratified, single arm, phase II trial.

Objective

The trial objective is to evaluate the therapeutic activity, safety and tolerability of axitinib in patients with advanced/metastatic soft tissue sarcoma who are unsuitable for or have relapsed after standard chemotherapy. The therapeutic activity will be separately assessed in angiosarcoma, synovial sarcoma, leiomyosarcomas and other sarcomas.

Sample size

Simon's two-stage design is applied separately to each patient group (stratum) and the trial requires a minimum of 72 patients and a maximum of 152 patients.

Trial duration

It is expected to complete accrual in 2 years and the trial in 3 years. All patients will be followed-up until death or a minimum follow-up period of one year.

Primary outcome measure:

- Progression-free survival rate at 12 weeks after starting treatment, defined according to RECIST criteria.

Secondary outcome measures:

- Tumour response rate (using RECIST criteria).
- Tumour response rate (using Choi criteria).
- Progression-free interval.
- Progression-free survival time.
- Overall survival time.
- Changes in performance status.
- Toxicity rate.

Biological measures

- Biomarkers of angiogenesis in blood and tumour biopsy samples.
- Assessment of CT scan based tumour texture as a biomarker of treatment response.

Eligibility criteria

Main (*but not exhaustive*) inclusion criteria

- Age ≥ 16
- Pathologically confirmed soft tissue sarcoma including angiosarcoma, leiomyosarcoma, synovial sarcoma and other eligible subtypes.
- Locally advanced or metastatic disease incurable by surgery or radiotherapy.
- Measurable disease according to RECIST criteria.
- Evidence of objective disease progression in the past 6 months, without anticancer treatment since progression.
- Patients ineligible for chemotherapy (e.g. through age, clinical condition or patient refusal) or who have received no more than two prior chemotherapy regimens.
- At least 4 weeks from prior anticancer treatment (surgery, radiotherapy and systemic therapies) and full recovery from all their adverse effects).
- WHO performance status 0, 1 or 2.

Main (*but not exhaustive*) exclusion criteria

- Ineligible pathological subtypes (defined in the protocol).
- Known central nervous system metastases.
- Previous malignancies (except curatively treated non-melanoma skin cancer or carcinoma in situ of the cervix or breast) within the past 3 years.
- Uncontrolled or poorly controlled hypertension.
- Heart failure ≥ NYHA class II.
- Thromboembolic events (arterial or venous thrombosis, myocardial infarction, unstable angina, cardiac angioplasty or stenting) within the past 12 months.
- Therapeutic dose warfarin.
- History of malabsorption or major gastrointestinal tract resection likely to affect trial drug absorption.
- Patients with cavitating lung metastases or any metatstasis abutting or invading a major pulmonary blood vessel on baseline CT or MRI scan.
- History of bleeding diathesis or coagulopathy within 12 months of study entry
- History within the previous 6 months of any blood clots in the sputum or streaky haemoptysis that was persistent (> 2 weeks) or recurrent (> 3 episodes).
- Regular treatment with antiplatelet medication, including aspirin >325 mg/day or NSAIDs.

Trial Schema

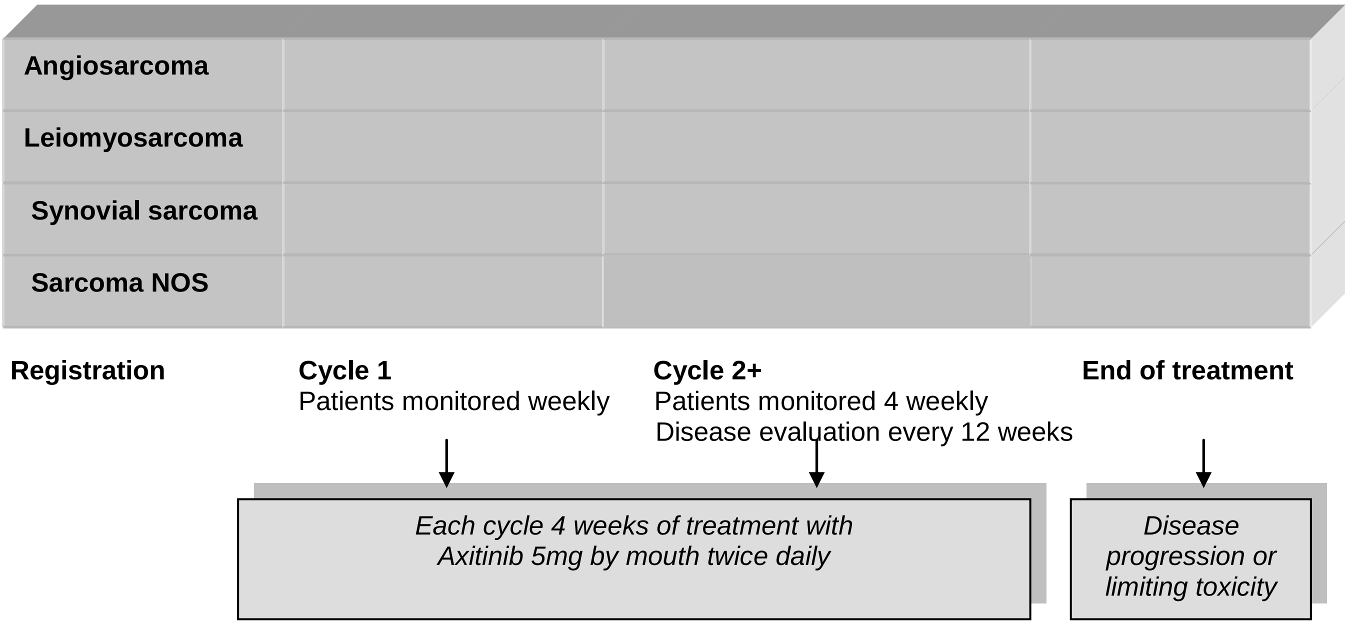

Chief Investigator details

Professor Penella Woll
Professor of Medical Oncology
University of Sheffield, Weston Park Hospital, Whitham Road, Sheffield S10 2SJ

Tel: 0114 226 5235 **Fax:** 0114 226 5364 **E-mail:** p.j.woll@shef.ac.uk

Trial Coordinator details

Ana Hughes
Cancer Research UK Clinical Trials Unit, School of Cancer Sciences, University of Birmingham, Edgbaston, Birmingham, B15 2TT

Tel: 0121 4143793 **Fax**: 0121 4142230 **E-mail**:a.i.hughes@bham.ac.uk

TRIAL MANAGEMENT GROUP

	Address	Telephone
**Chief Investigator**		
Prof. Penella Woll	Weston Park Hospital<br>University of Sheffield	0114 2265235
**Senior Trial Coordinator**		
Mrs. Ana Hughes	CR UK Clinical Trials Unit<br>University of Birmingham	0121 4143793
**Lead Biostatistician**		
Prof. Lucinda Billingham	CR UK Clinical Trials Unit<br>University of Birmingham	0121 4143790
**Trial Statistician**		
Mr. Piers Gaunt	CR UK Clinical Trials Unit<br>University of Birmingham	0121 4143410
**Pharmacist**		
Ms. Elizabeth Hodgkinson	Weston Park Hospital<br>University of Sheffield	0114 2265101
**Histopathologist**		
Dr. David Hughes	Royal Hallamshire Hospital<br>Sheffield	0114 2711900
**Radiologist**		
Dr Christine Ingram	Royal Hallamshire Hospital<br>Sheffield	0114 2713109
**Radiologist**		
Dr Irfan Kayani	University College London<br>Hospitals (UCLH)	0203 34567890

SPONSOR
Sheffield Teaching Hospitals NHS Foundation Trust
Research Department
11 Broomfield Road
Sheffield S10 2SE

TRIAL OFFICE
Mrs Ana Hughes, Trial Coordinator
Cancer Research UK Clinical Trials Unit
School of Cancer Sciences
University of Birmingham
Edgbaston, Birmingham B15 2TT
Tel: 0121 414 3793 Fax: 0121 414 2230
Email: a.i.hughes@bham.ac.uk
Office hours: Monday to Friday 9:00 a.m. to 5:00 p.m.

CLINICAL QUERIES
Clinical queries during office hours should be directed to the Chief Investigator
Professor Penella Woll
Tel: 0114 226 5235 Fax: 0114 226 5364
Email:p.j.woll@shef.ac.uk
or an appropriate member of the Trial Management Group

REGISTRATION
Cancer Research UK Clinical Trials Unit
University of Birmingham
Monday to Friday 9:00 a.m. to 5:00 p.m.
Tel: 0800 7317625 / 0800 371969
Fax: 0800 3286412

SAFETY REPORTING
Fax 0121 4142230 or 08003286412

TABLE OF CONTENTS

LIST OF ABBREVIATIONS AND DEFINITION TERMS 9
1. BACKGROUND 10
1.1 Soft tissue sarcoma 10
1.2 Angiosarcoma 10
1.3 Synovial sarcoma 11
1.4 Leiomyosarcoma 11
1.5 Axitinib 12
2. RATIONALE 12
3. PROPOSED PHASE III TRIAL IF THIS TRIAL IS POSITIVE 12
4. RISK/BENEFIT AND ETHICAL ASSESSMENT 13
5. OBJECTIVE AND OUTCOMES MEASURES 13
5.1 Objective 13
5.2 Outcome measures 13
5.3 Endpoints for stopping rules 14
5.4 Trial Design 14
6. PATIENT ELIGIBILITY 14
6.1. Site eligibility 14
6.2. Inclusion criteria 14
6.3. Exclusion criteria 15
7. WITHDRAWAL FROM TRIAL 16
8. PATIENT SCREENING, RECRUITMENT AND CONSENT 16
8.1. Patient Screening 16
8.2. Informed Consent 16
9. PATIENT ENROLMENT 17
10. TREATMENT DETAILS 17
10.1 Drug information 17
10.2 Pre-trial treatments 18
10.3 Duration of treatment 19
10.4 Expected toxicity 19
10.5 Dose and schedule modifications 20
10.6 Treatment compliance 21
10.7 Withdrawal from treatment 21
10.8 Concomitant medication 21
10.9 Post-trial treatment 21
11. PATIENT ASSESSMENTS 22
11.1 Blood pressure monitoring 22
11.2 Pre-treatment/screening assessments 22
11.3 On treatment assessments 22
11.4 End of treatment assessments 23
11.5 Follow-up assessments 23
11.6 Visit Schedule Table 23
12. SAFETY REPORTING 24
12.1 Definition of Adverse Event 24
12.2 Definition of Adverse Reaction 24
12.3 Definition of Unexpected Adverse Reaction 24
12.4 Definition of Serious Adverse Event 24
12.5 Definition of Serious Adverse Reaction (SAR) 25
12.6 Definition of Suspected Unexpected SAR (SUSAR) 25
12.7 List of Expected Adverse Reactions 25
12.8 Assessment of Adverse Events 25
12.9 Independent Assessment of Causality and Expectedness 26
12.10 Adverse Events Reporting 26
12.11 Expedited Serious Adverse Events 26
12.12 Overdose 26
12.13 Reporting by CRCTU 27
12.14 Data Monitoring Committee 27
13. DISCONTINUATION OF SUBJECTS FROM TREATMENT OR ASSESSMENT 28

13.1 Criteria for discontinuation 28
13.2 Procedures for discontinuation 28
14. CONFIRMATION OF RESPONSE 29
15. DEFINITIONS OF OUTCOME MEASURES 29
16. DATA HANDLING...30
16.1 Data Collection and Case Report Form 30
16.2 Data Management...30
16.3 Source Data..31
16.4 Archiving..31
16.5 Centralised Computerised Records 31
17. QUALITY MANAGEMENT..31
17.1 Site Set Up 31
17.2 On-site Monitoring 32
17.3 Central Monitoring 32
18. DEFINITION OF END OF TRIAL..32
19. STATISTICAL CONSIDERATIONS 32
19.1 Analysis of Primary Outcome Measure and Required Sample Size...32
19.2 Analysis of Secondary Outcome Measures 33
19.3 Translational Research 33
19.4 Timing of analysis..33
20. TRANSLATIONAL STUDIES...33
20.1 Methods 33
21. TRIAL ORGANISATIONAL STRUCTURE 33
21.1 Sponsor 33
21.2 Trial Management Group 33
21.3 Trial Steering Committee..33
21.4 Independent Data Monitoring Committee 34
22. ETHICAL AND LEGAL ASPECTS 34
22.1 Ethics Committee (EC) or Institutional Review Board (IRB) 34
22.2 Ethical Conduct of the Trial 34
22.3 Regulatory Authority Approvals /Authorisations..34
22.4 Patient Information and Consent..34
22.5 Patient's Rights to Withdraw 35
22.6 Confidentiality...35
23. FINANCE, INSURANCE AND INDEMNITY 36
23.1 Finance..36
23.2 Indemnity & Insurance..36
24. PUBLICATION & INTELLECTUAL PROPERTY RIGHTS 36
APPENDICES...37
Appendix 1: References 38
Appendix 2: NCI Common Terminology Criteria for Adverse Events..40
Appendix 3: Summary of RECIST 41
Appendix 4: WHO Performance Status Scale...44
Appendix 5: Cockcroft-Gault Formula 45
Appendix 6: Axitinib Clinical Interactions..46

LIST OF ABBREVIATIONS AND DEFINITION TERMS

The following abbreviations and special terms are used in this trial protocol.

Abbreviation or special term	Explanation
ALT	Alanine aminotransferase
ANC	Absolute Neutrophil Count
AST	Aspartate aminotransferase
bFGF	Basic Fibroblast Growth Factor
BP	Blood Pressure
CRCTU	Cancer Research UK Clinical Trials Unit
CT SCAN	Computerised Tomography scan
ECG	Electrocardiogram
ECHO SCAN	Echocardiogram Scan
EORTC	European Organisation for Research and Treatment of Cancer
GCP	Good Clinical Practice
INR	International Normalised Ratio
LVEF	Left Ventricular Ejection Fraction
MRI SCAN	Magnetic Resonance Imaging Scan
MUGA SCAN	Multiple Uptake Gated Acquisition Scan
NCRI	National Cancer Research Institute
NYHA	New York Heart Association
PCR	Polymerase Chain Reaction
PDGF	Platelet-Derived Growth Factor
PNET	Primitive Neuroectodermal Tumour
PPE	Palmar-Plantar Erythrodysesthesia
PT	Prothrombin Time
PTT	Partial Thromboplastin Time
QoL	Quality of Life
RBC	Red Blood Cell
RECIST	Response Evaluation Criteria In Solid Tumours
TNO	Trial Number
TSH	Thyroid Stimulating Hormone
VEGF	Vascular Endothelial Growth Factor
WBC	White Blood Cell
WHO	World Health Organisation

1. BACKGROUND

1.1 Soft tissue sarcoma

Soft tissue sarcomas are rare tumours accounting for 0.72% of new malignancies and 0.65% of malignant deaths[1]. They comprise a heterogeneous group of mesenchymal tumours including a wide variety of distinct pathological subtypes. These are usually grouped together for the purpose of treatment, but as knowledge of their biology emerges, it is expected that treatment will be specifically targeted at individual subtypes.

Although localised sarcomas can be cured by surgery with or without adjuvant radiotherapy, advanced sarcomas (recurrent or metastatic) carry a poor prognosis. First line chemotherapy is doxorubicin based, with response rates of about 20% and median survival of less than 12 months[2.] Combination chemotherapy is associated with a higher response rate and more toxicity, but no better survival[3]. Second line chemotherapy (with dacarbazine, ifosfamide or trabectedin) has lower response and survival rates[4]. There is an urgent need for better treatments.

In recent years, it has become apparent that the different subtypes of soft tissue sarcoma have specific molecular pathologies. Biologically targeted treatments promise to hold the key to their control. The exemplar for this is gastrointestinal stromal tumour (GIST), where treatment with tyrosine kinase inhibitors targeting mutated KIT (imatinib, sunitinib) have led to dramatic improvements in response rates and survival[5,6]. Angiogenesis, the process of new blood vessel formation is critical to the development of tumours larger than a few millimetres in diameter. There is some evidence that circulating levels of the angiogenic factors VEGF and bFGF are raised in patients with soft tissue sarcoma and correlated to disease extent and risk of recurrence[7,8]. There is also preliminary evidence of activity for a VEGFR inhibitor in synovial and other sarcomas[9]. Here we will explore the efficacy of a potent VEGFR inhibitor (axitinib) in soft tissue sarcomas, specifically angiosarcoma, synovial sarcoma and leiomyosarcoma.

1.2 Angiosarcoma

Angiosarcomas are rare malignant tumours with features of endothelial cell differentiation, which present as cutaneous or deep soft tissue masses[10]. Although most are sporadic, they can occur in association with radiation damage, chronic lymphoedema (Stewart-Treves syndrome), or inherited syndromes such as neurofibromatosis or Maffucci syndrome. Angiosarcoma is typically multifocal and has an insidious growth pattern, making local control difficult. The primary treatment is surgery, but local recurrence is common and the median survival 2-4 years[11, 12]. Radiotherapy can contribute to local disease control.

In patients with locally advanced or metastatic angiosarcoma, conventional chemotherapy with doxorubicin and ifosfamide has a response rate less than 20%[13]. Paclitaxel may be useful for scalp and facial angiosarcomas[14, 15]. A variety of other agents have been anecdotally reported to have success, including liposomal doxorubicin or daunorubicin[16, 17], docetaxel[18] and interleukin-2[19]. However, novel systemic therapies are clearly needed. A logical strategy is to investigate antiangiogenic therapies in this endothelial tumour. Most studies to date have focused on HIV-related Kaposi's sarcoma[20].

Angiosarcoma and related vascular tumours (eg. haemangioendothelioma, lymphangiosarcoma, Kaposi's sarcoma) characteristically express endothelial cell markers such as factor VIII-related antigen, CD34 (stem cell antigen), CD31 (endothelial cell surface antigen) and Ulex europeaus agglutinin-I (UEA-I). Data on the biology of angiosarcomas and the role of different vascular growth factors is sparse – most studies report expression of one or two factors in a very small series of tumour samples. No systematic study of the biology of angiosarcoma has been undertaken.

Expression of KIT has been demonstrated in both angiosarcoma cells and infiltrating mast cells[21]. Radiation-associated angiosarcomas can express KIT but molecular genetic analysis has shown no mutations in exon 11, suggesting that they will be resistant to imatinib[22]. Recent data have demonstrated expression of epidermal growth factor receptor (EGFR) and annexin-2 on angiosarcomas[23, 24]. The expression and role of vascular endothelial growth factor (VEGF) remains controversial[25, 26, 27]. Other angiogenic factors eg. basic fibroblast growth factor (bFGF), angiopoietins (Ang-1, -2 and -4), hypoxia inducible factor (HIF1α) and neuropilins which may play a role in these

vascular tumours have not been investigated[28, 29]. In addition there may be a role for endothelial precursor cells (EPC) in the development of angiosarcoma. EPCs (CD34+, CD45+, VEGFR2+) may reflect the phenotype of embryonic angioblasts, which are migratory endothelial cells with the capacity to circulate proliferate and differentiate into mature cells, do not mature into endothelium, but can be incorporated into the developing tumour microvasculature[30]. Both endothelial cells and EPC are increased in the circulation in cancer and may be a surrogate marker of treatment (ant-angiogenic) response[31, 32]. Interestingly, HIV-associated Kaposi sarcoma is attributable to co-infection with Kaposi sarcoma herpesvirus (KSHV) and has features of lymphatic endothelial cell differentiation, including regulation through VEGF-C (VEGFR-3) and angiopoietin-2 (Tie-2)[33, 34].

1.3 Synovial sarcoma

Synovial sarcoma represents 5%–10% of all sarcomas. It is more common in teenagers and young adults, and demonstrates a slight male predominance. The treatment of localised and metastatic disease is similar to that of other soft tissue sarcomas. For patients who relapse following primary treatment, the prognosis is poor. Specific t(X, 18) (p11.2; q11.2) translocations are associated with synovial sarcomas and are pathognomic for the tumour[35]. These translocations fuse the SYT gene on chromosome 18p11 and three of the six members of the SSX gene family on chromosome Xq11, namely, the SSX1 or SSX2 genes, and less frequently the SSX4 gene. The t(X, 18) translocation results in a gene encoding for a protein in which the addition of the C terminal domain of SSX to the SYT–SS18 protein affects its activation and repressive activities on gene expression.

Little is known of the expression of angiogenic factors in synovial sarcoma. A study of gene-expression patterns in synovial sarcoma using cDNA microarrays identified a unique pattern of expression of a cluster of 104 genes (r=0·66), which included synovial sarcoma X (SSX), retinoic acid pathway genes, and epidermal growth factor receptor but no angiogenesis-related genes[36]. Some activity has been seen with pazopanib, an anti-angiogenic tyrosine kinase inhibitor in synovial sarcoma[9]. This trial will test the efficacy of axitinib in synovial sarcoma and lead to systematic evaluation of angiogenic pathways in this tumour subtype.

1.4 Leiomyosarcoma

Leiomyosarcomas are relatively common soft tissue sarcomas and can arise in a wide variety of locations. Their behaviour and treatment is similar to that of other soft tissue sarcomas[37]. They have however shown responses to a wider range of chemotherapeutic agents including docetaxel, gemcitabine and trabectedin[38]. Because uterine leiomyosarcomas often express estrogen receptors, there is interest in using anti-estrogenic agents in these tumours.

Over expression of VEGF has been reported in leiomyosarcomas[7], but there is little other data on the expression of angiogenic factors in leiomyosarcoma. A study of gene-expression patterns in leiomyosarcoma using cDNA microarrays identified a distinct cluster of 24 genes (r=0·75) that was highly expressed in six of 11 leiomyosarcoma specimens. This group, part of a much larger cluster of genes, contained many genes known to be implicated in muscle structure and function, including actin, myosin, leiomodin, myosin phosphatase, and calponin[36]. Most of the other five leiomyosarcoma specimens did not express these markers, but were positive for desmin by immunohistochemistry. No angiogenesis-related genes were identified. Thalidomide is inactive in leiomyosarcoma, however some activity has been seen with bevacizumab[39] and pazopanib in leiomyosarcoma[9]. This trial will test the efficacy of axitinib in leiomyosarcoma and lead to systematic evaluation of angiogenic pathways in this tumour subtype.

1.5 Axitinib

Axitinib (AG-013736, Pfizer) is an orally bioavailable tyrosine kinase inhibitor targeting the VEGFR-1, VEGFR-2, VEGFR-3, PDGFR-ß and KIT receptors. VEGF receptors are critical components of the processes leading to the branching, extension and survival of endothelial cells which form new blood vessels during angiogenesis, a critical requirement for tumours to grow beyond microscopic size. *In vitro,* axitinib selectively blocks VEGF-stimulated receptor autophosphorylation, leading to inhibition of endothelial cell proliferation and survival. In receptor phosphorylation assays, the IC_{50} was 0.09-0.12nM for VEGFR-1, 0.20±0.06nM for VEGFR-2, 0.10-0.29nM for VEGFR-3, 1.6±0.4nM for PDGFR-ß and 1.70±0.6nM for KIT. It is not a potent inhibitor of the related tyrosine kinases FGFR-1, Flt-3, tie-2 or CSFR-1.

In mice, twice daily oral administration was well tolerated and inhibited tumour angiogenesis and the growth of colorectal, lung and islet cell tumours[40, 41]. Repeat-dose studies in mouse and dog of up to 26 weeks indicated effects on the gastrointestinal, haemopoietic, musculoskeletal and reproductive systems, which were reversible on stopping treatment. In mice, rats, dogs, monkeys and humans, axitinib was predominantly metabolised by CYP3A4 and has potential for clinical interactions with substrates of CYP2C8 and CYP1A2. In humans, axitinib metabolites are excreted in the faeces (37%) and urine (23%).

In a phase I study, 36 patients received axitinib at doses ranging from 5 to 30 mg by mouth twice daily[41]. The dose-limiting toxicities were hypertension, hemoptysis, and stomatitis but these were manageable at the recommended dose of 5mg bd. Axitinib was absorbed rapidly (bioavailablilty 58%), with peak plasma concentrations observed within 2 to 6 hours after dosing in the fasted state. No significant drug interaction with antacid was seen. Subsequent studies have shown similar pharmacokinetics in fed and fasting subjects, so it is now recommended that axitinib be given with food. Full details are provided in the Investigator brochure.

Clinical anti-tumour activity has been seen in renal cell cancer[42], thyroid cancer[43] and pancreas cancer (in combination with gemcitabine; [44]). Activity has also been observed in lung, breast and colorectal cancers, melanoma and other carcinomas. Safety data is available for 3595 subjects (see Investigator Brochure). Although frequent side effects have included fatigue, hypertension, diarrhoea, hand-foot syndrome and proteinuria, axitinib was well tolerated overall. Studies are ongoing in breast, colorectal, pancreas and lung cancer, AML and melanoma. It is inactive in acute myeloid leukemia or myelodysplastic syndrome[45].

2. RATIONALE

There is a clear requirement to identify new active agents in the treatment of soft tissue sarcoma. Angiogenesis is a hallmark of tumour growth, and there is increasing evidence that anti-angiogenic drugs, including axitinib, can lead to tumour regression and improved patient survival in a variety of tumours. This trial will evaluate the efficacy of single agent axitinib in soft tissue sarcomas, specifically including angiosarcoma, synovial sarcoma and leiomyosarcomas. Patients with advanced (incurable) disease who have received up to two prior chemotherapy regimens will be eligible. The stratified phase II trial design permits sites to recruit to each stratum in a single trial protocol, which should optimise recruitment in a multicentre UK-wide setting. By including systematic collection and study of biological samples for the evaluation of angiogenesis-related molecules, we will identify the pathways of angiogenesis used in these sarcoma subtypes.

3. PROPOSED PHASE III TRIAL IF THIS TRIAL IS POSITIVE

If this trial demonstrates activity of axitinib in any of the four strata, further investigation of this agent is warranted in a phase III trial in chemo-resistant disease. The best comparator may be best supportive care, paclitaxel, trabectedin or letrozole, depending on the stratum chosen. This could be undertaken as a UK or EORTC collaborative multi-centre trial. If the trial fails to demonstrate activity of axitinib in these sarcoma subtypes, the biological studies will permit rational planning of future studies using specific biologically targeted agents.

4. RISK/BENEFIT AND ETHICAL ASSESSMENT

It is recognised that patients joining this trial will expose themselves to a therapy of some toxicity and an investigation and visit schedule that carries a degree of burden of inconvenience and health risk. Set against this is the possibility of personal benefit based on a reasonable rationale and the common good achieved by the successful completion of a trial (whether positive or negative) in this little studied area.

5. OBJECTIVE AND OUTCOMES MEASURES

5.1 Objective

The objective of the trial is to evaluate the therapeutic activity, safety and tolerability of axitinib in patients with advanced/metastatic soft tissue sarcoma who have relapsed after standard chemotherapy. The therapeutic activity will be separately assessed in the eligible subtypes.

5.2 Outcome measures

Primary Outcome

Progression-free survival rate at 12 weeks after starting treatment, defined according to RECIST criteria

Secondary Outcomes

- Tumour response rate (using RECIST criteria)
- Tumour response rate (using Choi criteria)
- Progression-free interval
- Progression-free survival time
- Overall survival time
- Changes in performance status
- Toxicity rate

Biological measures

Blood, fresh, frozen and paraffin-embedded tumour biopsy material will be collected for independent review of the pathological diagnosis and the study of biomarkers of angiogenesis, which will be correlated with response and survival data.

A paraffin-fixed block will be requested from relevant histopathology departments for the pathological and biological studies, including mutational analysis. Up to 20 spare 4 micron sections will need to be cut up. If sufficient tissue is available, a further block should be sent from which tissue microarray cores can be extracted. Original blocks will be returned to the originating pathology departments.

For angiosarcomas only, fresh tumour material will be required. If not already available, a core biopsy should be taken prior to trial entry whenever possible. The studies to be undertaken include the following:

- Immunohistochemistry for factor VIII-related antigen, CD34, CD31, UEA-I, KIT, PDGFR and VEGFR-1, -2, -3, Tie-2, PDGF and VEGF ligands, angiopoietins 1,2,4, neuropilin NP1 and 2, bFGF, Ki67, EPCs.
- Molecular genetic analysis for mutated p53, KIT and VEGFR.

Analysis of CT scan based tumour texture as a biomarker of treatment response. The analysis will be performed centrally at UCLH. This will be done using dedicated analysis software.
Only the anonymised CT scans collected as part of the central review of responses will be sent to UCLH. All CT scans will be provided on CD and stored securely to ensure patient confidentiality.

5.3 Endpoints for stopping rules

Although axitinib is an unlicensed medicinal product, it has been evaluated in over 750 patients in over 20 clinical trials, and considerable experience of the adverse event profile has been obtained. Serious adverse events from all axitinib studies will be monitored by the Trial Management Group (TMG) in real time. If any serious unexpected toxicities emerge with significant frequency, the trial will be stopped.

5.4 Trial Design

The trial is an open label, multicentre, stratified, single arm, phase II trial, using the Simon's two-stage design, with pathological and molecular genetic analysis of tumour samples. This trial design has been used successfully by the EORTC Soft Tissue & Bone Sarcoma Group to evaluate new agents. The drug will be separately investigated in 4 tumour subtypes:

- Angiosarcoma
- Synovial sarcoma
- Leiomyosarcoma (uterine, skin or non organ origin)
- Other types of eligible soft tissue sarcoma

Simon's two-stage design is applied separately in each stratum. 18 or 33 eligible patients will be registered in each stratum, according to the number of responses seen in the first 18 patients. Patients will be evaluated after 12 weeks treatment. Those with stable or responding disease and no limiting toxicity may continue treatment until disease progression.

The trial requires a minimum of 72 patients and a maximum of 152 patients (allowing for 5 extra patients on each stratum to cover ineligible and non-treated patients).

Patients will be recruited from major sarcoma centres in the UK, with approximately 15 investigative sites participating in the trial.

6. PATIENT ELIGIBILITY

6.1. Site eligibility

To participate, a site must complete a feasibility questionnaire prior to application for Site Specific Approval (SSA). The site must be able to comply with the protocol and data collection requirements. All patients must be reviewed by a sarcoma Multi-Disciplinary Team. Treatment must be managed in a dedicated oncology facility under the supervision of an oncology consultant supported by an oncology research team familiar with GCP requirements and the safety profile and characteristics of axitinib.

6.2. Inclusion criteria

a) Pathologically confirmed soft tissue sarcoma, including:
   - Angiosarcoma, including intermediate and malignant vascular tumours (WHO classification, 2002) and Kaposi's sarcoma.
   - Leiomyosarcoma, including uterine, skin or non organ origin.
   - Synovial sarcoma.
   - Other eligible subtypes of soft tissue sarcoma of Trojani intermediate or high grade, including fibroblastic, fibrohistiocytic, adipocytic, rhabdomyosarcoma, malignant peripheral nerve sheath, and NOS. See exclusion criteria for ineligible subtypes.

b) Locally advanced or metastatic disease incurable by surgery or radiotherapy.
c) Measurable disease according to RECIST criteria.
d) Evidence of objective disease progression in the past 6 months, without anticancer treatment since progression.
e) Patients ineligible for chemotherapy (eg. through age, clinical condition or patient refusal) or who have received no more than two prior chemotherapy regimens.
f) Age $\geq$ 16.
g) WHO performance status 0, 1 or 2.
h) At least 4 weeks from prior anticancer treatment (surgery, radiotherapy and systemic therapies) and full recovery from all their adverse effects.

i) No evidence of preexisiting uncontrolled hypertension as documented by 2 baseline blood pressure readings taken at least 1 hour apart. The baseline systolic blood pressure readings must be ≤140 mm Hg, and the baseline diastolic blood pressure readings must be ≤90 mm Hg. Patients whose hypertension is controlled by antihypertensive therapies are eligible.
j) Adequate physiological function:
   - renal : calculated or measured creatinine clearance ≥ 50 ml/min using the Cockcroft-Gault formula (see appendix 5).
   - haematological: ANC ≥ 1.5 x 10^9/L, platelets ≥ 100 x 10^9/L, INR ≤ 1.2.
   - hepatic: bilirubin within normal range, AST and ALT ≤ 3 x upper limit of normal.
   - cardiac: LVEF (measured by ECHO or MUGA) within normal range.
   - Urinary protein <2+ by urine dipstick. If dipstick is ≥2+ then a 24-hour urine collection can be done and the patient may enter only if urinary protein is <2 g per 24 hours.
k) Negative pregnancy test and agrees to comply with contraceptive measures.
l) Able to swallow oral medication.

6.3. Exclusion criteria

a) Ineligible pathological subtypes including:
   - Osteosarcoma
   - Ewings/PNET sarcomas
   - Chondrosarcoma
   - Gastrointestinal stromal tumours (GIST)
   - Dermatofibrosarcoma protuberans (DFSP)
   - Malignant mesothelioma
   - Mixed mesodermal tumours of uterus
b) Known central nervous system metastases.
c) Age < 16.
d) Current use or anticipated need for treatment with drugs that are known CYP3A4 or CYP1A2 inducers (i.e., carbamazepine, dexamethasone, felbamate, omeprazole, phenobarbital, phenytoin, primidone, rifabutin, rifampicin, and St. John's Wort).
e) Current use or anticipated need for treatment with drugs that are known potent CYP3A4 inhibitors (i.e., grapefruit juice, verapamil, ketoconazole, miconazole, itraconazole, voriconazole, erythromycin, clarithromycin, ergot derivatives, indinavir, saquinavir, ritonavir, nelfinavir and lopinavir).
f) Previous malignancies (except curatively treated non-melanoma skin cancer or carcinoma in situ of the cervix or breast) within the past 3 years.
g) Heart failure ≥ NYHA class II.
h) Patients with cavitating lung metastases or any metatstasis abutting or invading a major pulmonary blood vessel on baseline CT or MRI scan.
i) History of bleeding diathesis or coagulopathy within 12 months of study entry
j) History within the previous 6 months of any blood clots in the sputum or streaky haemoptysis that was persistent (> 2 weeks) or recurrent (> 3 episodes).
k) Any of the following within the 12 months prior to trial drug administration: myocardial infarction, severe/unstable angina, coronary/peripheral artery bypass graft, symptomatic congestive heart failure, cerebrovascular accident or transient ischemic attack, deep vein thrombosis or pulmonary embolism.
l) Therapeutic dose warfarin. Low molecular weight heparin is permitted.
m) Regular treatment with antiplatelet medication, including aspirin >325 mg/day or NSAIDs.
n) History of malabsorption or major gastrointestinal tract resection likely to affect trial drug absorption.
o) Pregnancy or breastfeeding. Female patients must be surgically sterile or be postmenopausal, or must agree to use two effective contraception measures during the period of therapy which should be continued for 4 weeks after the last dose of trial therapy. Male patients must be surgically sterile or must agree to use effective contraception during the period of therapy which

should be continued for 4 weeks after the last dose of trial therapy . The definition of effective contraception will be based on the judgment of the Investigator or designee.

7. WITHDRAWAL FROM TRIAL

In the event of a patient's decision to withdraw from the trial, the Investigator should ascertain from which aspects of the trial the patient wishes to withdraw (see below) and details should be recorded in the Case Report Form (CRF).

(a) If the patient chooses to withdraw from treatment only, he or she should discontinue treatment and continue to be assessed in accordance with protocol.
(b) If the patient wishes to withdraw from the trial (i.e. including trial specific assessments), then no more trial specific assessments will be performed. However, the patient should be followed up for disease progression and for survival status unless consent for this is withdrawn explicitly. This information should be recorded in the CRF. All Serious Adverse Events should be reported until 28 days after last administration of axitinib.

NOTE: All information and tissue samples collected up until the point of retraction will be retained and analysed. Withdrawn patients will not be replaced as all patients will continue to be followed by the NHS Information Centre, Medical Research Information Service (MRIS).

8. PATIENT SCREENING, RECRUITMENT AND CONSENT

8.1. Patient Screening

Investigators will be expected to maintain a Screening Log of all potential trial candidates (i.e. those who have received a Patient Information Sheet). This Log will include limited information about the potential candidate (i.e. date of birth and gender), date, and outcome of the screening process (e.g. enrolled into trial, reason for ineligibility, or refused to participate).

For patients who appear to meet the criteria for participation in the trial, the Investigator will provide information to allow them to make an informed decision regarding their participation. If informed consent is given, the Investigator will conduct a full screening evaluation to ensure that the patient satisfies all inclusion and exclusion criteria. A patient who gives written informed consent and who satisfies all the inclusion and exclusion criteria may be entered into the trial.

8.2. Informed Consent

It is the responsibility of the Investigator or designee to obtain written informed consent for each patient prior to trial entry and prior to performing assessments that are not conducted as standard of care. The approved trial Patient Information Sheet must be used in this process.

The Investigator or designee must ensure that they adequately explain the aims, trial treatment, trial schedule, anticipated benefits and potential hazards of taking part in the trial to the patient. The Investigator or designee should also stress that the patient is completely free to refuse to take part or withdraw from the trial at any time. The patient should be given ample time (at least 24 hours) to read the Patient Information Sheet and to discuss their participation with others outside of the research team. The patient must be given an opportunity to ask questions which should be answered to their satisfaction. The right of the patient to refuse to participate in the trial without giving a reason must be respected.

If the patient expresses an interest in participating in the trial they should be asked to sign and date the latest version of the Informed Consent Form in the presence of the Investigator or designee who must then co-sign and date the form. The patient must personally initial all boxes. A copy of the Informed Consent Form should be given to the patient, a copy should be filed in the hospital notes, and the original placed in the Investigator Site File (ISF). A dated annotation should be made in the patient medical notes confirming eligibility and stating that consent has been taken.

Electronic copies of the Patient Information Sheet and Informed Consent Form will be made available to sites and should be printed or photocopied onto the headed paper of the local institution. With the patient's prior consent, their General Practitioner (GP) will also be informed that they are taking part in the trial. A GP Letter is provided electronically for this purpose.

Throughout the trial, the patient should have the opportunity to ask questions about the trial and any new information that may be relevant to the patient's continued participation should be shared with them in a timely manner. On occasion it may be necessary to re-consent the patient in which case the process above should be followed and the patient's right to withdraw from the trial respected.

9. PATIENT ENROLMENT

Once patients have signed the Informed Consent Form, and their eligibility has been confirmed through the screening assessments, a registration form should be completed and the details should be phoned or faxed through to the Cancer Research UK Clinical Trials Unit (CRCTU), Birmingham, between 9:00 a.m. to 5:00 p.m. Monday to Friday.

☎: 0800 731 7625 or 0800 371 969
Fax: 0800 328 6412

At the end of the registration procedure the patient will be allocated with a unique Trial Number (TNO). The TNO should be recorded on prescriptions for trial medication. Trial medication should not be dispensed in the absence of a valid TNO.

The TNO should be recorded on the registration form and this should be signed and dated and sent to the trial office. A copy of the registration form should be filed in the Investigator Site File.

10. TREATMENT DETAILS

10.1 Drug information

Identity of investigational product

The investigational product in this trial is axitinib (AG-013736, N-methyl-2-[3-((E)-2-pyridin-2-yl-vinyl)-1H-indazol-6-ylsulfanyl]-benzamide, IUPAC).

Doses and treatment schedules

Patients will take axitinib tablets 5 mg by mouth twice daily continuously. There may be one dose reduction to 3 mg twice daily. A four week dosing period will be considered as 1 cycle of treatment. Axitinib treatment will be continued until disease progression, or the development of limiting toxicity.

Drug supply/distribution

Axitinib is being provided free of charge. Pfizer will supply axitinib 3mg and 5mg tablets to Sharp Clinical Services (previously known as Bilcare GCS (Europe) Ltd). Sharp Clinical Services will be responsible for distributing to sites. Initial supplies of axitinib will be sent out to sites by Sharp Clinical Services after they have been informed by the CRCTU that all approvals are in place. Subsequent supplies should be ordered by pharmacy using the form provided. Fax the completed form to Sharp Clinical Services.

On receipt of axitinib, details should be completed on the Shipment Form and faxed back to Sharp Clinical Services and the Shipment Form filed in the Pharmacy File.

If supplies are damaged on arrival please contact Sharp Clinical Services and inform the CRCTU. Damaged supplies should be disposed of on site according to local policy and a certificate of disposal completed and placed in the Pharmacy File.

Copies of the dispatch requests, shipment forms and certificate(s) of analysis will be held with the site and/or CRCTU for the required period of time.

Storage and stability

The pharmacist will ensure that all investigational product is stored in a secured area, under recommended storage conditions and in accordance with applicable regulatory requirements. Drug should be stored at controlled room temperature between 15-30°C avoiding exposure to light. The trial drug must not be used outside the context of this protocol. Under no circumstances should the Investigator or other site personnel supply trial drug to other investigators, patients, or clinics, or allow supplies to be used other than directed by this protocol without prior authorisation from the Sponsor.

Handling

Axitinib is a hazardous drug (due to possible reproductive toxicity), and should be handled according to the recommended procedures described in each institution's pharmacy or hospital standard operating procedure manual for handling hazardous drugs.

Dispensing

Axitinib will be dispensed at the beginning of Week 1 (Day 1) and thereafter every 4 weeks. In case of dose modification, patients will be requested to return all of their previously dispensed unused medication to the clinic, and they will be dispensed new ones.

Drug accountability

The pharmacy must maintain adequate records documenting the receipt, use, loss, or other deposition of the investigational product. CRCTU will supply pharmacy with a drug accountability form or may approve use of the standard institution form. In either case, the forms must identify the investigational product, including batch or code numbers, and account for its disposition on a patient-by-patient basis, including specific dates and quantities. The forms must be signed by the individual who dispensed the drug and copies must be provided to CRCTU. The prescribed dose should also be recorded in the patient's medical records.

Administration

Patients will take axitinib tablets by mouth twice daily continuously for the duration of the trial. The starting dose is 5 mg twice daily approximately 12 hours apart and approximately at the same time each day. The tablets should be taken with food.

If a patient vomits after taking a dose, the patient should not "make it up". Any missed doses may be taken late up to 3 hours before the next scheduled dose, otherwise it should be skipped. If doses are missed or vomited, this must be indicated in the source documents and CRF's.

Patient monitoring

Patients will be monitored once weekly for cycle 1, then at 4 week intervals. Toxicity (including hypertension) will be closely monitored. Disease progression will be evaluated every 12 weeks. Axitinib treatment will be continued until disease progression, or development of limiting toxicity.

Patient returns

Patient returns of axitinib should be returned to pharmacy for counting and recording in the patient's Dispensing Log. Patient returns may then be disposed of on site according to local hospital policy (verification by a CRCTU trial monitor is not required).

Drug disposal

Expired drug or drug left unused at the end of the trial should be disposed of at site according to local hospital policy. A dated certificate of disposal should be completed. The original certificate should be placed in the Pharmacy File and a copy faxed to the CRCTU.

Action in case of overdose

Treat symptoms developed by the patient.

10.2 Pre-trial treatments

See eligibility, section 6.

10.3 Duration of treatment

Patients may continue to receive treatment with axitinib until disease progression or development of limiting toxicity.

10.4 Expected toxicity

For single agent axitinib, the most common treatment related all causality adverse events reported include:

Incidence > 40%	Incidence 20%-40%	Incidence 10%-20%
• Fatigue<br>• Diarrhoea<br>• Hypertension	• Anorexia<br>• Nausea<br>• Hoarseness or dysphonia<br>• Palmar-plantar erythrodysaesthesia (PPE)<br>• Weight loss<br>• Vomiting<br>• Hypothyroidism<br>• Cough<br>• Constipation	• Dyspnoea<br>• Stomatitis<br>• Arthralgia<br>• Proteinuria<br>• Headache<br>• Extremity pain<br>• Rash<br>• Mucositis<br>• Back pain<br>• Dyspepsia<br>• Abdominal pain<br>• Dizziness

The most commonly reported treatment-related adverse events of at least grade 3 are hypertension (20.1%), fatigue (10.1%) and diarrhoea (9.0%).

In ongoing studies of single agent axitinib, the most commonly reported laboratory adverse events of at least grade 3 were: lymphocytes (8.5%), hyperglycaemia (4.8%), hyponatraemia (4.8%), lipase (4.5%), hypoalbuminaemia (2.9%), proteinuria (2.9%), AST (2.9%), hypophosphataemia (2.9%), haemoglobin (2.6%), amylase (2.3%), ALT (2.2%) and hyperkalaemia (2.0%). Of note, neutropenia (1.1%) and thrombocytopenia (0.3%) were very uncommon.

Cardiac failure events

In clinical studies with Axitinib for the treatment of patients with RCC, cardiac failure events (including cardiac failure, cardiac failure congestive, cardiopulmonary failure, left ventricular dysfunction and right ventricualr failure) were reported in 1.8% of the patients receiving axitinib. Grade 3/4 cardiac failure events were reported in 1.0% of the patients and fatal cardiac failure events were reported in 0.3% of the patients receiving axitinib.

Monitor for signs or symptoms of cardiac failure periodically throughout treatment with axitinib. Management of cardiac failure events may require temporary interruption or permanent discontinuation and/or dose reduction of axitinib therapy.

Haemorrhage has been associated with the use of compounds that inhibit VEGFR or bind VEGF (e.g. bevacizumab). Tumour-related haemorrhage may occur suddenly, and in the case of pulmonary tumours, may present as severe and life-threatening haemoptysis or pulmonary haemorrhage. For example, in patients with non-small cell lung cancer, two episodes of fatal haemoptysis occurred in 114 patients treated with axitinib either alone (n=32) or in combination with chemotherapy (n=82). One episode of fatal pulmonary haemorrhage occurred in the first 50 sarcoma patients recruited to the Axi-STS trial.Two distinct patterns of bleeding have occurred in subjects receiving axitinib. The first is minor haemorrhage, most commonly NCI-CTCAE Grade 1 epistaxis. The second is serious, and in some

cases fatal, haemorrhage. During the axitinib development programme, haemorrhagic Serious Adverse Events have included gastrointestinal haemorrhage, cerebral haemorrhage, haemoptysis, epistaxis, rectal haemorrhage, intracranial haemorrhage and subarachnoid haemorrhage. If haemorrhage occurs, treatment should be withheld until the symptoms are medically managed per local standard of care and the patient is stabilized.

Further, less common adverse effects are detailed in the Axitinib Investigator Brochure. Axitinib is an unlicensed compound. Unexpected adverse effects may occur and must be reported to the trial office.

10.5 Dose and schedule modifications

Toxicity

In the event of **any grade 3 toxicity** or worse, axitinib treatment should be discontinued until the toxicity has recovered to grade 1 or better, when axitinib can be reintroduced **at a lower dose** of 3 mg twice daily. Treatment may be interrupted for a maximum of 2 weeks. If the toxicity has not improved sufficiently within this time, the patient should be withdrawn from the trial. Dose and schedule modifications for specific adverse events are listed below:

Hypertension

Hypertension is very common and should be actively managed with medication. An angiotensin converting enzyme inhibitor (eg. Ramipril 1.25 mg) or calcium channel blocker (eg, amlodipine 5 mg) is recommended as initial treatment.
If two BP readings separated by at least 1 hour show the following:

- Systolic blood pressure (SBP) of 151-160 mm Hg OR
- Diastolic blood pressure (DBP) of 91-105 mm Hg

then new or additional antihypertensive therapy should be started while continuing with full-dose axitinib.

If two BP readings separated by at least 1 hour show the following:

- SBP >160 mm Hg OR
- DBP >105 mm Hg

Then axitinib should be discontinued until the blood pressure has been controlled with medication.

Treatment may be interrupted for a maximum of 2 weeks. Treatment with axitinib should then be restarted at a reduced dose of 3 mg as soon as the SBP reduces to < than150 mm Hg and the DBP reduces to < than 90 mm Hg.

If the blood pressure has not improved sufficiently within this time (ie. To ≤ 150/90), the patient should be withdrawn from the trial.

Haemoptysis

Treatment with axitinib will be temporarily discontinued for haemoptysis of >½ teaspoon (2.5 mL) of bright red blood per day. Such patients should be assessed by chest x-ray or CT/MRI scan for evidence of cavitating lesions (see below). If the haemoptysis resolves to baseline within 1 week and there is no evidence of disease cavitation, treatment with axitinib may continue at the current dose level or be decreased by one dose level at the discretion of the treating investigator. Patients who experience haemoptysis without resolution to baseline within 1 week or who have evidence of disease cavitation should discontinue treatment with axitinib.

Cavitating lung metastases

Severe haemoptysis has been seen in patients developing cavitation in lung metastases while taking axitinib. Chest x-ray or CT/MRI scan will be performed at 4, 8 and 12 weeks and 12 weekly thereafter to assess for cavitation. Any patient developing cavitation in lung metastases should immediately be withdrawn from the trial.

Proteinuria

- If dipstick shows > 1+ proteinuria, perform 24 hour urine collection. Dosing may continue while waiting for test results.
- If < 2 g proteinuria/24 hour is reported, continue dosing at the same dose level.
- If ≥ 2 g proteinuria/24 hours is reported, hold dosing and repeat 24 hour urine collection for proteinuria and creatinine clearance (interval at investigator discretion) until proteinuria is < 2 g/24 hours. Restart axitinib at the same dose or one lower dose level at discretion of the investigator.

Thrombocytopenia
Patients should discontinue axitinib for **grade 3** thrombocytopenia (platelets <50 x 10^9/L) until the platelet count is >50, then axitinib can be reintroduced **at a lower dose** of 3 mg twice daily. Treatment may be interrupted for a maximum of 2 weeks. If the toxicity has not improved sufficiently within this time, the patient should be withdrawn from the trial.

10.6 Treatment compliance

Patients will be required to return all bottles of trial medication at the end of each 4 week period. The number of tablets remaining will be documented and recorded. Patients will be asked to estimate how many doses were missed during the previous 4 weeks. Patients whose compliance falls below 50% of intended adherence will be considered to have met a direct indication for withdrawal from further treatment.

10.7 Withdrawal from treatment

Any of the following constitutes a direct indication to withdraw the patient from further treatment with axitinib:

1. Clear evidence of generalised disease progression
2. Intolerable toxicity
3. Patient refusal
4. > 14 day dose delay
5. < 50% compliance over a 4 week period
6. If the managing physician feels that further treatment would not be in the patient's best interest

10.8 Concomitant medication

No other chemotherapy, radiotherapy, or experimental anticancer medications will be permitted while the patient is on trial.

Patients who need to be on anticoagulant therapy during treatment with axitinib should be treated with low molecular weight heparin as the preferred therapy.

Patients should not take antiplatelet drugs, including aspirin (>325mg/day) and NSAIDs.

Axitinib is not likely to have drug-drug interactions with commonly used antihypertensive agents belonging to the class of ACE inhibitors including angiotensin II receptor antagonists (enalapril, captopril, losartan, valsartan), beta-blockers (atenolol, metoprolol, labetalol), or diuretics (hydrochlorothiazide, furosemide). Within the class of calcium channel blockers, verapamil, and to a lesser extent nifedipine, nicardipine, and diltiazem have a potential for increasing axitinib plasma concentrations due to CYP3A4 inhibition and should not be used as first choice in antihypertensive treatment. Other calcium channel blockers (amlodipine, bepridil, felodipine) are less likely to raise axitinib plasma levels.

10.9 Post-trial treatment

After completion of trial treatment, further anti-tumour treatment with alternative agents will be at the discretion of the Investigator, but must be recorded in the CRF.

11. PATIENT ASSESSMENTS

11.1 Blood pressure monitoring

Patients treated with axitinib will be issued blood pressure cuffs for home monitoring and instructed to measure their blood pressure (BP) twice daily, prior taking each dose. All blood pressure measurements will be recorded in a diary and brought to the nurse or investigator at each clinic visit. Patients should be instructed by the trial staff to contact their clinician immediately for guidance if their systolic blood pressure rises above 150 mm Hg, diastolic blood pressure rises above 90 mmHg, or if they develop symptoms perceived to be related to elevated BP (headache, visual disturbance).

11.2 Pre-treatment/screening assessments

Within 4 weeks prior to starting trial treatment, the following are required:

- Disease assessment by CT or MRI scan (for evaluable disease and evidence of disease progression in the 6 months prior to trial entry). For cutaneous disease, clinical photographs, including a ruler, will be required.

Within 2 weeks prior to starting trial treatment, the following are required:

- Medical history and physical examination (including cancer symptoms and signs, body weight, WHO performance status, pulse rate and blood pressure).
- Blood tests for:
    - Full blood count including Hb, WBC, neutrophils & platelets.
    - Clotting studies including INR.
    - Biochemistry including, urea, creatinine, total protein, albumin, electrolytes, total bilirubin, alkaline phosphatase, gamma-GT, AST (SGOT) or ALT (SGPT).
    - Thyroid function tests – T4 and TSH.
    - Store serum/plasma samples for VEGF.
- Urinary protein (by urine dipstick)
- Chest x-ray (This can be omitted if patient has a chest x-ray on Day 1 of treatment)
- 12-lead ECG
- Cardiac ECHO or MUGA scan
- Pregnancy test
- Concomitant medication
- Tumour samples for translational studies

11.3 On treatment assessments

Assessments will be performed weekly during cycle 1, then at the start of each 4-week treatment cycle.

- Physical examination (cycle 2+)
- Performance status
- Weight
- Pulse rate
- Blood pressure
- Full Blood count
- Urinary protein (by urine dipstick)
- Biochemistry
- Thyroid function (cycle 2 +)
- Serum/plasma VEGF (cycle 2 +)
- Concomitant medication
- Chest X-Ray at 4 weeks, 8 weeks, 12 weeks and every 12 weeks thereafter, except when a CT or MRI scan done at the same time point includes the whole lungs.
- Tumour assessment by CT / MRI scan and photos, where indicated (12 weekly)
- Toxicity

11.4 End of treatment assessments

The end-of-treatment assessments should be completed within 28 days of the last axitinib dose:

- Physical examination
- Performance status
- Weight
- Pulse rate
- Blood pressure
- Full Blood count
- Biochemistry
- Thyroid function
- Serum/plasma VEGF
- Concomitant medication
- Chest X-Ray (not necessary if already done to show PD)
- Tumour assessment by CT/MRI scan and photos, where indicated (not necessary if already done to show PD)
- Toxicity

11.5 Follow-up assessments

Disease evaluation should be carried out 12 weeks after trial entry (even if the trial treatment has already been discontinued) then every 12 weeks until disease progression. After disease progression, patients should be followed up every 3 months for survival.

11.6 Visit Schedule Table

	Screening	Cycle 1 weekly	Cycles 2+ 4 weekly	End of treatment
History	✓			
Physical examination	✓		✓	✓
Performance status	✓	✓	✓	✓
Weight	✓	✓	✓	✓
Pregnancy Test	✓			
Pulse rate & Blood pressure	✓	✓	✓	✓
Full Blood count	✓	✓	✓	✓
Urinary protein	✓	✓	✓	
Clotting studies	✓			
Biochemistry	✓	✓	✓	✓
Thyroid function	✓		✓	✓
Serum VEGF	✓		✓	✓
Tumour for translational studies	✓			
Concomitant meds.	✓	✓	✓	✓
Chest X-Ray	✓		4, 8, 12 weeks and 12 weekly thereafter except when a CT or MRI scan done at the same time	✓ not necessary if already done to show PD

			point includes the whole lungs.	
Tumour assessment by CT / MRI scan and photos	✓		12 weekly	✓ not necessary if already done to show PD
ECG & cardiac ejection fraction ECHO or MUGA scan	✓			
Toxicity		✓	✓	✓

12. SAFETY REPORTING

12.1 Definition of Adverse Event

An Adverse Event (AE) is defined as any untoward medical occurrence in a patient or clinical trial patient administered trial treatment and which does not necessarily have a causal relationship with the treatment received.
Comment:-*An AE can therefore be any unfavourable and unintended sign (including abnormal laboratory findings), symptom or disease temporally associated with the use of the trial treatment, whether or not it is considered related to the trial treatment.*

12.2 Definition of Adverse Reaction

An Adverse Reaction (AR) is defined as all untoward and unintended responses to trial treatment related to any dose administered.
Comment: *An AE judged by either the reporting Investigator or Sponsor’s Safety/Clinical coordinator as being possibly related, probably related, or definitely related to trial therapy will qualify as an AR.*

12.3 Definition of Unexpected Adverse Reaction

An Unexpected Adverse Reaction (UAR) is defined as an AR, the nature or severity of which is not consistent with the applicable product information).

Comment: *When the outcome of an AR is not consistent with the applicable product information the AR should be considered unexpected.*

12.4 Definition of Serious Adverse Event

A Serious Adverse Event (SAE) is defined as any untoward medical occurrence or effect that at any dose:

- Results in death
- Is life-threatening*
- Requires inpatient hospitalisation** or prolongation of existing hospitalisation
- Results in persistent or significant disability or incapacity
- Is a congenital anomaly/birth defect
- Or is otherwise considered medically significant by the Investigator (i.e. an event that jeopardises the patient or may require intervention to prevent one of the other outcomes listed above)

* *Life threatening in the definition of an SAE refers to an event in which the patient was at risk of death at the time of the event; it does not refer to an event that hypothetically might have caused death if it were more severe.*

** *Hospitalisation is defined as an unplanned, formal inpatient admission, which may or may not involve an overnight period, even if the hospitalisation is a precautionary measure for continued observation. Thus hospitalisation for protocol treatment (e.g. line insertion), elective procedures (unless*

brought forward because of worsening symptoms) or for social reasons (e.g. respite care) are not regarded as an SAE.

Comment: *The term severe is often used to describe the intensity (severity) of a specific event. This is not the same as serious, which is based on patients/event outcome or action criteria.*

12.5 Definition of Serious Adverse Reaction (SAR)

A Serious Adverse Reaction (SAR) is a SAE judged by either the reporting Investigator or Sponsor's Safety/Clinical Coordinator as being possibly related, probably related, or definitely related to trial therapy.

12.6 Definition of Suspected Unexpected SAR (SUSAR)

A Suspected Unexpected Serious Adverse Reaction (SUSAR) is defined as a SAR that is unexpected i.e. the nature, seriousness, severity (i.e. grade) or outcome of the event is not consistent with the applicable product information.

12.7 List of Expected Adverse Reactions

For a list of all expected adverse reactions please refer to the Investigator's Brochure.

12.8 Assessment of Adverse Events

For all AE's the Investigator will determine the grade (i.e. severity), seriousness and causality (i.e. relationship to trial therapy) as described below.

Grade (i.e. severity)
All events should be graded according to the NCI CTCAE Toxicity Criteria (Version 4.0).

For events not listed in the toxicity table, severity should be recorded as:

Mild	Does not interfere with patients usual functioning
Moderate	Interferes to some extent with patient usual functioning
Severe	Interferes significantly with patients usual functioning
Life threatening	Risk of death, organ damage or disability

Causality (i.e. Relationship to trial therapy)
Relationship to trial therapy will be assessed using the following definitions:

Unrelated	There is no evidence of any causal relationship.
Unlikely to be related	There is little evidence to suggest there is a causal relationship (e.g. the event did not occur within a reasonable time after administration of the trial drug). There is another reasonable explanation for the event (e.g. the patient's clinical condition, other concomitant treatments).
Possibly related	There is some evidence to suggest a causal relationship (e.g. the event occurred within a reasonable time after administration of the trial drug). However, the influence of other factors may have contributed to the event (e.g. the patient's clinical condition, other concomitant events).
Probably related	There is evidence to suggest a causal relationship, and the influence of other factors is unlikely.

Definitely related	There is clear evidence to suggest a causal relationship, and other possible contributing factors can be ruled out.

12.9 Independent Assessment of Causality and Expectedness

Seriousness and causality of all reported SAE's will be determined independently by the Local and Chief Investigators. An SAE judged by the Local or Chief Investigator to have a reasonable causal relationship with the trial drug will be regarded as a SAR (i.e. possibly related, probably related, or definitely related to trial therapy). The Chief Investigator will also assess all SAR's for expectedness. If the event meets the definition of a SAR that is unexpected in nature it will be classified as a SUSAR.

12.10 Adverse Events Reporting

Investigators or designee should report all SAE's in an expedited manner (i.e. within 24 hours of becoming aware of the event) as outlined below.

The Investigator should assess the seriousness of all AE's experienced by the patient. AE's defined as serious and which require reporting as a SAE (see excluded events listed below) should be reported on a SAE Form. When completing the form the Investigator will be asked to define the causality (relatedness) of the event to the trial intervention, and the severity of AE which should be documented using the NCI CTCAE Toxicity Criteria, version 4.0 (contained in the Investigator Site File).

On becoming aware that a patient has experienced an SAE, the Investigator or designee must complete, date and sign a SAE Form. The form should be faxed together with a SAE Fax Cover Sheet, to the trial office using one of the numbers listed below as soon as possible and no later than 24 hours after first becoming aware of the event:

Fax: 0121 4142230 or 08003286412

Receipt of SAE's will be acknowledged by the trial office.

If the SAE Form is completed by someone other than the Investigator, the Investigator will be required to countersign the original SAE Form, to confirm that they agree with the causality and severity assessments. The form should then be returned to the trial office in the post and a copy kept in the ISF.

Investigators should also report SAE's to their own Trust in accordance with local practice.

Investigators should give details of all AE and SAE's from date of patients enrolled into trial until 28 days after the last exposure to the trial treatment.

Patients should be followed up until resolution of the event.

12.11 Expedited Serious Adverse Events

In the case of an SAE the Investigator must immediately:

- Complete a SAE Form (If more than one SAE occurs, each event will be recorded separately on a new form) and a fax cover sheet.
- The SAE Form can be completed and signed by a member of the site trial team delegated this responsibility by the Investigator, but should be checked and counter signed by the local Investigator at a later date.
- Report SAE in accordance with local institutional policy.
- Continue to follow the patient up until clinical recovery is complete or any sequelae has stabilised.
- Provide follow-up information on an SAE Form on resolution of the event.

12.12 Overdose

Overdose is classed as an SAE and would need to be managed as such.

12.13 Reporting by CRCTU

Reporting is in line with the “Detailed guidance on the collection, verification and presentation of adverse reaction reports arising from clinical trials on medicinal products for human use April 2006”.

For the purposes of the trial axitinib is classed as Investigational Medicinal Products (IMP).

SUSAR reporting

The CRCTU shall report all individual events which are unexpected and suspected to be related to axitinib to the competent authority and the ethics committee as follows:

If SUSAR is categorised as a fatal or life threatening SUSAR, a minimal data set of all individual events will be sent within 7 days. With detailed follow-up information provided within an additional 8 days.
All other events categorised as SUSAR’s will be reported within 15 days to the competent authority and the ethics committee in the concerned Member State.

Annual Safety report

In addition to the above, the CRCTU shall submit, once a year throughout the clinical trial or on request, a safety report to the competent authority and the ethics committee, taking into account all new available safety information received during the reporting period. The annual safety report will describe concisely all new safety information relevant to assess the safety conditions of patients included in the concerned trial.

Investigators

Details of all SUSAR’s and any other safety issue which arises during the course of the trial will be compiled into a report and sent to all Investigators by the CRCTU. The frequency of reporting will be dependent on the volume of SUSAR’s generated and the significance of the safety issue but will be at least every 6 months. The line listing will present data on all SUSAR’s.

Pfizer

CRCTU will report to Pfizer by fax any SAE that occurs in a trial subject during the event reporting period within 24 hours of first awareness of the event (immediately if the event is fatal or life-threatening).

Other Safety Issues Identified During the Course of the Trial

The CRCTU shall notify the competent authority and the ethics committee immediately if a significant safety issue is identified during the course of the trial.

12.14 Data Monitoring Committee

An independent Data Monitoring Committee will review all SAE’s.

12.15 Pregnancy Reporting

Any pregnancy or fathering of a child during treatment and up to 4 weeks after the last axitinib intake has to be recorded using a Clinical Trial Pregnancy Form and reported by the Investigator to the CRCTU.

To ensure patient safety, each pregnancy in a patient on trial drug must be reported to the CRCTU within 24 hours of learning of its occurrence. The pregnancy should be followed up to determine outcome, including spontaneous or voluntary termination, details of the birth, and the presence or absence of any birth defects, congenital abnormalities, or maternal and/or newborn complications.

Pregnancy follow-up should be recorded on the same form and should include an assessment of the possible relationship to the trial drug of any pregnancy outcome. Any SAE experienced during pregnancy must be reported on the SAE Form.

If the father is taking the trial drug, informed consent to report information regarding pregnancy outcome needs to be obtained from the mother.

12.16 Laboratory safety measurements and variables reporting

The sites will perform laboratory analyses according to the Visit Schedule (see section 11.5). The trial office must be provided with a copy of the laboratory's certification, and a tabulation of the normal ranges for each parameter required. If at any time a patient has laboratory parameters obtained from a different outside laboratory the normal ranges for that laboratory should be recorded with the result obtained.

At any time during the trial, abnormal laboratory parameters which are clinically relevant (e.g. require dose modification and/or interruption of trial drug, lead to clinical symptoms or signs or require therapeutic intervention), whether specifically requested in the protocol or not, must be recorded on the appropriate CRF page. When abnormal laboratory values or test results constitute an adverse event (i.e., induces clinical signs/symptoms or requires therapy) they must be recorded on the Adverse Events CRF.

Haematology

Haematology includes assessment of haemoglobin, hematocrit, RBC, total WBC count, platelet count, and a differential count including neutrophils, lymphocytes, monocytes and eosinophils, basophils. If the patient experiences any abnormal internal bleeding, the coagulation profile (analyses of bleeding time, platelet aggregation, and PT and PTT measurements) will be explored and abnormality will be recorded on the Adverse Events CRF.

Biochemistry

Biochemistry includes urea, creatinine, total protein, albumin, total bilirubin, alkaline phosphatase, gamma-GT, AST (SGOT) or ALT (SGPT).

Vital signs, ECG, blood pressure, heart rate and physical examination

A physical examination including vital signs will be performed according to the Visit Schedule. Information about the physical examination and vital signs must be present in the source documentation at the trial site. Significant findings present prior to the start of trial drug must be included in the relevant CRF. Significant findings made after the start of trial drug which meet the definition of an adverse event must be recorded on the Adverse Events CRF.

Performance status / Body weight

Measurements of performance status and body weight will be performed according to the visit schedule. The WHO Performance Status Scale will be used in this trial.

Dosage Administration record

The total daily dose of axitinib will be recorded in milligrams. Any change in dose or interruption in daily dose administration must be recorded on the case report form.

13. DISCONTINUATION OF SUBJECTS FROM TREATMENT OR ASSESSMENT

13.1 Criteria for discontinuation

See section 10.7

13.2 Procedures for discontinuation

Patients with a global deterioration of health status requiring discontinuation of treatment without objective evidence of disease progression at that time should be reported as "symptomatic deterioration". Every effort should be made to document the objective progression even after discontinuation of treatment.

14. CONFIRMATION OF RESPONSE

The main goal of confirmation of objective response is to minimise the risk of overestimation of the response rate. In cases where confirmation of response is not feasible, it should be made clear when reporting the outcome of such studies that the responses are not confirmed.

To be assigned a status of Partial Response (PR) or Complete Response (CR), changes in tumour measurements must be confirmed by repeat assessments that should be performed no less than 4 weeks after the criteria for response are first met.

In the case of Stable Disease (SD), follow-up measurements must have met the SD criteria at least once after trial entry at 12 weeks.

Responses will be centrally reviewed. Investigators must undertake to supply anonymised scans and photos to the trials unit for response assessment.

15. DEFINITIONS OF OUTCOME MEASURES

Progression-free survival rate at 12 weeks after starting treatment

Disease will be assessed by CT or MRI scan 12 weeks after entry to trial and will be compared to disease measured by CT or MRI scan on entry to the trial or within 4 weeks prior to entry. Response at 12 weeks will be measured using RECIST criteria. Progression-free survival rate is measured as the number of patients who are alive and progression-free at 12 weeks divided by the total number of patients who received at least one cycle of treatment.

Tumour response rate

Disease will be assessed at 12 weeks by CT or MRI scan and clinical photographs where indicated, and compared with disease measured at baseline. Response will be measured using RECIST criteria. Tumour response rate is defined as the number of patients who achieved a complete or partial response divided by the total number of patients who received at least one cycle of treatment.

Progression-free interval

Progression-free interval is defined as the time from trial entry to date when disease progression first observed where progression is defined in terms of RECIST criteria. Patients who die of disease without disease progression being recorded are defined as progression at date of death. Those that die of other causes are censored at date of death and those who are alive with no recorded progression at time of analysis are censored at date last seen alive and progression-free.

Progression-free survival time

Progression-free survival time is defined in the same way as progression-free interval except that all deaths, whatever the cause, are included as events in the analysis.

Overall survival time

Survival time is measured from trial entry to death from any cause or to date last seen alive for those patients who are still alive at the time of analysis.

Change in performance status

Changes in performance status from baseline according to the WHO performance status scale.

Toxicity rate

Adverse events will be graded using NCI Common Terminology Criteria for Adverse Events (CTCAE) v4.0. Toxicity rate is defined as the number of patients experiencing at least one grade 3, 4 or serious adverse reaction divided by the total number of patients who started treatment.

16. DATA HANDLING

16.1 Data Collection and Case Report Form

The Investigator or designee will record data as accurately and completely as possible on each patient, as soon as the requested information is available. The Investigator will be responsible for the timing, completeness, legibility and accuracy of the patient's Case Report Form (CRF) and he/she will retain a copy of each completed form. The Investigator will supply the Sponsor's representative with any required background data from such records.

Entries should be made in pen and black or blue ink on the CRF provided and must be legible. Any errors should be crossed out with a single stroke, the correction inserted and the change initialled and dated by the Investigator or designee. If it is not clear why a change has been made, an explanation should be written next to the change. Typing correction fluid should not be used.

Data reported on CRF should be consistent with the source data or the discrepancies should be explained. When data is directly entered into the CRF, the entry in CRF will be considered as source data.

If information is not known, this must be clearly indicated e.g. by entering Not Known (NK) on the form as missing and ambiguous data will be queried.

The CRF may be amended during the course of the trial; this will not constitute a protocol amendment. Revised CRF will be circulated to participating sites with immediate effect; sites should ensure they are using the most recent version of the CRF.

The completed originals should be sent to the trial office, with a copy held by the Investigator at site in a secure location (e.g. patient notes, secure filing cabinet).

16.2 Data Management

Monitoring will be carried out according to the CRCTU Monitoring Plan.
Monitoring and auditing procedures defined/agreed by the Sponsor will be followed, in order to comply with Good Clinical practice (GCP) Guidelines. All sites that recruit 3 or more patients will be visited to ensure compliance with trial protocol, GCP and legal aspects. Visits to other recruiting sites will be at the discretion of the TMG.

Returned CRF's will be checked for missing or unusual values (range checks), timing and consistency over time. If any such problems are identified during in house monitoring, a Data Clarification Form (DCF) specifying the problematic form within CRF will be created and sent to sites. The correct data should be written on the DCF and signed by responsible person at the site (as assigned on the responsibilities form). The complete DCF should be returned to the trial office and the site should retain a copy of the DCF with the relevant page of the CRF.

Sites may be suspended from further recruitment in the event of serious and persistent non-compliance and/or very poor recruitment.

Recording of data/retention of records

The Investigator or designee is responsible for completing the CRF's in a timely manner and clarifying and resolving any data queries. The completed and corrected CRF's for completed visits will be sent to the trial office. A copy of the CRF's will be retained by the Investigator, who must ensure that it is stored with other trial documents.in a secure place.

Data on patients collected on CRF's during the trial will be documented in an anonymous fashion and the patient will only be identified by the trial number, initials, date of birth, and hospital name and/or number. If, as an exception, it is necessary for safety or regulatory reasons to identify the patient, both the CRCTU and the Investigator are bound to keep this information confidential.

All the information required by the protocol should be provided and any omissions require explanation. All CRF's must be completed and available for collection in a timely manner after the patient's visit.

The Investigator must maintain source documents for each patient in the trial. All information on CRF's must be traceable to these source documents, which are generally maintained in the patient's file. The source documents should contain all demographic and medical information, including laboratory data, electrocardiograms, etc., also a copy of the signed informed consent form, which should indicate the trial number and title of the trial.

The Investigator, as listed below, must retain essential documents, as long as needed to comply with national and international regulations. The Investigator agrees to adhere to the document retention procedures by signing the protocol. Essential documents include:

1. IRB/IEC approvals for the trial protocol and all amendments
2. All source documents and laboratory records
3. CRF copies
4. Patients informed consent forms
5. Any other pertinent trial document

16.3 Source Data

Source data is all the information in original records and certified copies of original records of clinical findings, observations, or other activities in the trial, which are necessary for the reconstruction and evaluation of the trial. In the following cases the CRF will be considered the source document: - Quality of Life; pain questionnaires etc.

16.4 Archiving

It is the responsibility of the Investigator to ensure all essential trial documentation and source records (e.g. signed informed consent forms, investigator site files, pharmacy files, patients' hospital notes, copies of CRF's etc.) at their site are securely retained for at least 5 years after the end of the trial. Participating sites will be sent a letter specifying the permissible disposal date.

16.5 Centralised Computerised Records

Details of sites and participating staff will be recorded during the trial onto computer system at the CRCTU. Records of sites and participating staff will be modified to maintain accurate details of personnel and status held on the computer system.

Data from CRF will be modified to correct any erroneous or missing entries. The reason for these changes will be recorded in an audit trail.

At the conclusion of the trial i.e. when all patient data has been collected, and the analysis is complete, all the data stored on the CRCTU computer system will be archived for 15 years after the completion of the trial. After trial conclusion, if any audit is required or new analysis to be performed, the data will be retrieved.

17. QUALITY MANAGEMENT

The trial is being conducted under the auspices of the CRCTU according to the current guidelines for Good Clinical Practice (GCP). Participating sites will be monitored by CRCTU staff to confirm compliance with the protocol and the protection of patients' rights as detailed in the Declaration of Helsinki.

17.1 Site Set Up

All sites will be required to sign a Clinical Trial Site Agreement prior to participation. In addition all participating investigators will be asked to sign other necessary agreements and supply a current C.V. to the trial office. All members of the site research team will also be required to sign the Site Signature and Delegation Log, which should be returned to the trial office. Prior to commencing recruitment all sites will undergo a process of initiation. Key members of the site research team will be required to attend either a meeting or a teleconference covering aspects of the trial design, protocol procedures, adverse event reporting collection, and reporting of data and record keeping. Sites will be provided with an Investigator

Site File and a Pharmacy File containing essential documentation, instructions, and other documentation required for the conduct of the trial. The trial office must be informed immediately of any change in the site research team.

17.2 On-site Monitoring

Monitoring will be carried out as required following a risk assessment and as documented in the CRCTU Quality Management Plan. Additional on-site monitoring visits may be triggered by poor CRF return, poor data quality, excess toxicity, excessive number of patient withdrawals or deviations. If a monitoring visit is required the trial office will contact the site to arrange a date for the proposed visit and will provide the site with written confirmation. Investigators will allow the trial staff access to source documents as requested.

17.3 Central Monitoring

Trials staff will be in regular contact with the site research team to check on progress and address any queries that they may have. Trials staff will check incoming case report forms for compliance with the protocol, data consistency, missing data and timing. Sites will be sent data clarification forms requesting missing data or clarification of inconsistencies or discrepancies.

Sites may be suspended from further recruitment in the event of serious and persistent non-compliance with the protocol and/or GCP, and/or poor recruitment. Any major problems identified during monitoring may be reported to the Trial Management Group, Trial Steering Committee and the relevant regulatory bodies. This includes reporting serious breaches of GCP and/or the trial protocol to the main REC and the Medicines for Health Care products Regulatory Agency (MHRA).

18. DEFINITION OF END OF TRIAL

The end of the trial is defined as the date of the last study visit of the last patient undergoing the trial. All patients will be followed up for a minimum of one year.

19. STATISTICAL CONSIDERATIONS

19.1 Analysis of Primary Outcome Measure and Required Sample Size

The trial is based on Simon's two-stage phase II minimax design, with each patient group (stratum) analysed separately. The analysis of progression-free survival rate (PFR) in phase II trials of active and inactive agents by the EORTC Soft Tissue & Bone Sarcoma Group (van Glabbeke et al, 2002) showed that for second-line therapy, a 3-month PFR of ≥ 40% suggests drug activity, and ≤ 20% suggests inactivity. The primary outcome measure for the trial is therefore chosen as progression-free survival rate at 12 weeks after the start of treatment, such that patients with stable or responding disease at this time are defined as a success. For each stratum, success in 40% or more (P1) is considered worthwhile for further study, whereas success in 20% or less (P0) is considered unacceptable. The trial is designed such that there is a 5% chance of incorrectly accepting axitinib as worthy of further investigation (significance level) and 80% chance of correctly detecting that axitinib is worthy of further investigation (power). Based on these criteria, if 11 successes are seen in 33 eligible treated patients (followed for 12 weeks), the drug is deemed active in this stratum. To avoid treating too many patients with an inactive drug, an analysis will be performed in the first 18 patients. If less than 5 successes are seen, then this stratum will be closed, but if 5 or more successes are seen, then the trial will continue to recruit the full 33 patients. Assessment at this first stage requires a minimum of 12 weeks follow-up for the first 18 patients but recruitment will not be suspended as stopping does not relate to a safety outcome. It is anticipated that approximately 4 patients per strata may be recruited into the trial during this stage 1 follow-up period. Up to 5 patients may be added to allow for ineligible or untreated patients, giving a maximum of 38 patients per stratum.

Across all strata the trial requires a minimum of 72 and a maximum of 152 patients. If 10 UK centres participate, it should be possible to complete accrual in 2 years and complete the trial in 3 years.

19.2 Analysis of Secondary Outcome Measures

Each stratum will be analysed separately. Progression-free interval, progression-free survival time and overall survival time will be analysed using the Kaplan-Meier method of estimation. Curves will be plotted and medians and rates at 1 year will be estimated with 95% confidence intervals. Response and toxicity rates will be reported with 95% confidence intervals and all adverse events experienced and their associated grades will be reported descriptively.

19.3 Translational Research

Responses and survival will be correlated with expression of vascular growth factor receptors.

19.4 Timing of analysis

As specified in 19.1, an interim analysis of each stratum will take place after 18 eligible treated patients have completed 12 weeks of follow-up. At this interim analysis, if 5 or more patients are observed to be alive and progression-free then the trial will continue to recruit to a final target of 33 patients per stratum. The final analysis of the primary outcome measure will take place after all patients have been followed for a minimum of 12 weeks and final analysis of all outcome measures will take place after all patients have been followed up for a minimum of 1 year.

20. TRANSLATIONAL STUDIES

The translational studies are an intrinsic part of the protocol, not an optional extra. Blood samples will be taken for estimation of circulating angiogenic factors, including serum VEGF at trial entry and every cycle. Paraffin-fixed and fresh-frozen tumour samples will be studied for a wide range of biomarkers, in addition to independent review of the pathological diagnosis. For angiosarcomas only, if fresh-frozen tumour is not available, a core biopsy should be taken whenever possible prior to trial entry. The objective is to determine whether biomarkers of angiogenesis are predictive for response to axitinib, or for prognosis. In addition, new targets for biologically directed treatments may be identified.

See manual for collection of samples contained in the Investigator Site File.

20.1 Methods

Serum VEGF will be monitored using ELISA assays for VEGF-A, -B and –C. We will undertake a systematic study of fresh and fixed sarcoma samples for growth factor expression. Immuno-histochemistry will be used for endothelial cell markers (eg. factor VIII-related antigen, CD34, CD31, CD105, UEA-I, robo-4), endothelial growth factors and their receptors (eg. KIT, PDGFR and VEGFR-1, -2, -3, Tie-2, PDGF and VEGF ligands, angiopoietins 1,2,4, neuropilin NP1 and 2, bFGF) and the proliferation marker, Ki67. We also plan to undertake PCR analysis of mutated KIT, VEGFR and EGFR in DNA extracted from the tumour specimens. Tissue microarrays will be constructed from tumour blocks. Samples will be retained for future proteomic and genomic analysis.

21. TRIAL ORGANISATIONAL STRUCTURE

21.1 Sponsor

Sheffield Teaching Hospitals NHS Foundation Trust.

21.2 Trial Management Group

The Trial Management Group (TMG) will be responsible for the day-to-day running and management of the trial and will meet by teleconference or in person as required.

21.3 Trial Steering Committee

A Trial Steering Committee (TSC) will provide overall supervision for the trial and provide advice through its independent chair. The ultimate decision for the continuation of the trial lies with the TSC. The TSC will meet at least once a year or more often if required.

21.4 Independent Data Monitoring Committee

An independent Data Monitoring Committee (DMC) has been established for this trial. The Trial statistician and DMC will be the only group who sees the confidential, accumulating data to the trial. Their main objective will be to advise the TSC as to whether there is any evidence or reason as to why the trial should be amended or terminated based on the recruitment rates or safety.

Reports to the DMC will be produced by the Statistician and Trial Coordinator. The committee will meet one year after the trial opens and then annually thereafter until the trial closes to recruitment or more often if requested by the DMC. The DMC may consider discontinuing the trial if the recruitment rate or data quality are unacceptable or if the trial compromises patient safety in any way.

22. ETHICAL AND LEGAL ASPECTS

22.1 Ethics Committee (EC) or Institutional Review Board (IRB)

Documented approval from appropriate ECs/IRBs will be obtained for all participating sites prior to start of trial in respective sites, according to GCP, local laws, regulations and organisations. When necessary, an extension, renewal or amendments must be obtained from appropriate ECs/IRBs and also forwarded to Sponsor.

The ECs must supply to the Sponsor, upon request, a list of the EC members involved in the vote and statement to confirm that the EC is organised and operates to GCP and applicable laws and regulations.

22.2 Ethical Conduct of the Trial

The procedures set out in the protocol, pertaining to the conduct, evaluation, and documentation of the trial are designed to ensure that the Sponsor and investigator abide by GCP guidelines and under the guiding principles detailed in the Declaration of Helsinki. The trial will also be carried out in keeping with applicable local laws and regulations. This may include an inspection by the Sponsor ‘s representatives and/or regulatory authority representatives at any time.

The investigator must agree to the inspections of trial–related records by Regulatory Authority/Sponsor’s representatives, and must allow direct access to source documents to regulatory authorities/Sponsor’s representatives.

Any variation in procedure from that specified in the protocol may lead to the results of the trial being questioned and in some cases rejected. Any proposed protocol change must therefore be submitted in writing to the Sponsor to be pre-approved by the Sponsor. However the investigator may implement a deviation from, or a change of the protocol to eliminate an immediate hazard (s) to the trial subjects without prior EC/IRB/Sponsor approval/favourable opinion. As soon as possible, the implemented deviation or change, the reasons for it and if appropriate the proposed protocol amendment should be submitted to EC/IRB/Sponsor. Any deviations from the protocol must be fully explained and documented by the investigator.

22.3 Regulatory Authority Approvals /Authorisations

Regulatory Authority approvals/authorisations/notifications, where required, must be in place and fully documented prior to trial start.

22.4 Patient Information and Consent

It is the responsibility of the Investigator to obtain written informed consent from each patient prior to entering the trial or, where relevant, prior to evaluating the patient's suitability for the trial. For details of informed consent procedure see section 8.2.

A patient information sheet and informed consent form will be provided. Prior to the beginning of the trial, the investigator must have ECs/IRB written approval/favourable opinion of the written informed consent form and any other written information provide to subjects. The written approval of the EC/IRB

together with the approved patient information/informed consent forms must be filed in the Investigator Site File.

Written informed consent must be obtained before any trial specific procedure takes place. Participation in the trial and date of consent should be documented appropriately in patient's files.

22.5 Patient's Rights to Withdraw

A patient has the right to withdraw from the trial without giving reasons and without prejudicing his/her further treatment. See section 7 for more details regarding withdrawal.

22.6 Confidentiality

The personal data recorded on all documents will be regarded as strictly confidential and will be handled and stored in accordance with the applicable laws and/or regulations.

To preserve the patient's anonymity, the unique trial number, their initials, date of birth, and hospital name and/or number will be recorded on the case report forms. If the subject name appears on any documents (e.g. pathologist report) it must be obliterated before a copy of the document is supplied to the trial office.

The Investigator must ensure the patient's anonymity is maintained. The Investigator must maintain documents not for submission to the trial office in strict confidence.
The trial office will maintain the confidentiality of all patient data and will not reproduce or disclose any information by which patients could be identified. Patients should be reassured that their confidentiality will be respected at all times.

Trial findings stored on a computer will be stored in accordance with local data protection laws. The patients will be informed that representatives of the Sponsor EC/IRB, or regulatory authorities may inspect their medical records to verify the information collected, and that all personal information made available for inspection will be handled in strictest confidence and in accordance with local data protection laws.

When results are published, the patient's identity will remain confidential. The Investigator will maintain a log to enable patients' records to be identified.

In the UK, with the patient's permission, their name and NHS number will be collected at registration to allow flagging with the Medical Research Information Service (MRIS).

The personal data recorded on all documents will be regarded as strictly confidential and will be handled and stored securely in accordance with the Data Protection Act, 1998. The patient's full name, date of birth, National Health Service (NHS) number, or in Scotland the Community Health Index (CHI), and hospital number will be collected at trial entry to allow tracing through the MRIS and to assist with long-term follow-up via other health care professionals (e.g. patient's GP). However, patients will be identified using only their unique trial number, initials, hospital number and date of birth on any correspondence between the trial office and the participating site.

The Investigator must maintain documents not for submission to the trial office (e.g. Patient Identification Log) in strict confidence. In the case of special problems and/or governmental queries, it will be necessary to have access to the complete trial records, provided that patient confidentiality is protected.

The trial office will maintain the confidentiality of all patient data and will not disclose information by which patients may be identified to any third party, other than those directly involved in the treatment of the patient. Representatives of the trial office may be required to have access to patient's hospital records for quality assurance purposes but patients should be reassured that their confidentiality will be respected at all times.

23. FINANCE, INSURANCE AND INDEMNITY

23.1 Finance

The trial is funded by CTAAC and Pfizer.
Axitinib is provided free of charge by Pfizer.

23.2 Indemnity & Insurance

This trial is a clinician-initiated and clinician-led trial sponsored by Sheffield Teaching Hospitals NHS Foundation Trust.

No provision has been made for indemnity in the event of a claim for non-negligent harm.

In terms of liability, NHS Trust and Non-Trust Hospitals have a duty of care to patients treated, whether or not the patient is taking part in a clinical trial. Compensation is only available via NHS indemnity in the event of clinical negligence being proven.

24. PUBLICATION & INTELLECTUAL PROPERTY RIGHTS

Results of this trial will be submitted for publication in a peer reviewed journal. The manuscript will be prepared by the Trial Management Group (TMG); The Chief Investigator and Trial Statistician will be senior authors, and the Trial Pathologist and investigators registering at least 10% of the eligible patients will be among the co-authors. Any secondary publications and presentations must be reviewed by the TMG. Manuscripts must be submitted to the TMG in a timely fashion, in advance of being submitted for publication to allow time for review and resolve any outstanding issues. Authors must acknowledge that the trial was performed with the support of Sheffield Teaching Hospitals NHS Trust, Cancer Research UK, the University of Birmingham and Pfizer.

To ensure against inadvertent disclosure of unprotected inventions that relate to the Pfizer Product, the Chief Investigator will provide Pfizer an opportunity (a minimum of 30 days before submission or other public disclosure) to prospectively review any proposed publication, abstract, or other type of disclosure that reports the results of the trial. Pfizer shall respond as early as possible and in any case within 20 days of receipt of the proposed publication. Chief Investigator will comply with Pfizer requests for revisions to prevent inadvertent disclosure of Pfizer confidential information. Pfizer may make recommendations for other revisions to publications which will be made at the discretion of the Chief Investigator.

Intellectual Property rights will be addressed in the agreements between Sponsor and sites.

APPENDICES

Appendix 1: References

Appendix 2: NCI Common Terminology Criteria for Adverse Events

Appendix 3: Summary of RECIST

Appendix 4: WHO performance Status

Appendix 5: Cockcroft-Gault Formula

Appendix 6: Axitinib clinical interactions

Appendix 1: References

1. Jemal A, Siegel R, Ward E, et al. Cancer Statistics 2008. CA Cancer J Clin 2008; 58: 71-96.
2. Santoro A, Tursz T, Mouridsen H, et al. Doxorubicin versus CYVADIC versus doxorubicin plus ifosfamide in first-line treatment of advanced soft tissue sarcomas: a randomized study of the EORTC Soft Tissue & Bone Sarcoma Group. J Clin Oncol 1995; 13: 1537-1545.
3. Bramwell VH, Anderson D, Charette ML. Doxorubicin-based chemotherapy for the palliative treatment of adult patients with locally advanced or metastatic soft-tissue sarcoma: a meta-analysis and clinical practice guideline. Sarcoma. 2000;4(3):103-12.
4. Van Glabbeke M, Verweij J, Judson I, et al. Progression-free rate as the principal end-point for phase II trials in soft-tissue sarcomas. Eur J Cancer 2002; 38: 543-9.
5. Verweij J, Casali PG, Zalcberg J, et al. Progression-free survival in gastrointestinal stromal tumours with high-dose imatinib: randomised trial. Lancet 2004; 364: 1127–134.
6. Demetri GD, van Oosterom AT, Garrett CR, et al. Efficacy and safety of sunitinib in patients with advanced gastrointestinal stromal tumour after failure of imatinib: a randomised controlled trial. Lancet 2006; 368: 1329-38.
7. Potti A, Ganti AK, Foster H, et al. Immunohistochemical detection of HER-2/neu, c-kit (CD117) and vascular endothelial growth factor (VEGF) overexpression in soft tissue sarcomas. Anticancer Res 2004; 24: 333-7.
8. Yoon SS, Segal NH, Olshen AB, et al. Circulating angiogenic factor levels correlate with extent of disease and risk of recurrence in patients with soft tissue sarcoma. Ann Oncol 2004; 15: 1261-6.
9. Sleijfer S, Papai Z, Le Cesne A, et al. Phase II study of pazopanib (GW786034) in patients (pts) with relapsed or refractory soft tissue sarcoma (STS): EORTC 62043. J Clin Oncol 2007; 25 (18S): 10031.
10. Weiss SW, Lasota J, Miettinen MM. Angiosarcoma of soft tissue. In: Fletcher CDM, Unni KK, Mertens F (Eds). WHO Classification of Tumours. Pathology and genetics of tumours of soft tissue and bone. IARC Press, Lyon, 2002: 175-177.
11. M Fayette J, Martin E, Piperno-Neumann S, et al. Angiosarcomas, a heterogeneous group of sarcomas with specific behavior depending on primary site: a retrospective study of 161 cases. Ann Oncol 2007; 18: 2030-2036.
12. Meis-Kindblom JM, Kindblom LG. Angiosarcoma of soft tissue: a study of 80 cases. Am J Surg Pathol. 1998; 22: 683-97.
13. Budd GT. Management of angiosarcoma. Curr Oncol Rep. 2002; 4: 515-9.
14. Casper ES, Waltzman RJ, Schwartz GK, et al. Phase II trial of paclitaxel in patients with soft-tissue sarcoma. Cancer Invest. 1998;16:442-6.
15. Fata F, O'Reilly E, Ilson D, et al. Paclitaxel in the treatment of patients with angiosarcoma of the scalp or face. Cancer 1999; 86:2034-7.
16. Lankester KJ, Brown RS, Spittle MF. Complete resolution of angiosarcoma of the scalp with liposomal daunorubicin and radiotherapy. Clin Oncol, 1999; 11: 208-10.
17. Wollina U, Fuller J, Graefe T, et al. Angiosarcoma of the scalp: treatment with liposomal doxorubicin and radiotherapy. J Cancer Res Clin Oncol 2001; 127: 396-9.
18. Nagano T, Yamada Y, Ikeda T, et al. Docetaxel: A therapeutic option in the treatment of cutaneous angiosarcoma: report of 9 patients. Cancer 2007; 110: 648-51.
19. Kojima K, Okamoto I, Ushijima S, et al. Successful treatment of primary pulmonary angiosarcoma. Chest 2003; 124: 2397-400.
20. Heymach JV. Angiogenesis and antiangiogenic approaches to sarcomas. Curr Opinion Oncol 2001;13:261-9.
21. Yamamoto T, Umeda T, Nishioka K. Immonohistological distribution of stem cell factor and kit receptor in angiosarcoma. Acta Derm Venereol 2000; 80:443-5.
22. Komdeur R, Hoekstra HJ, Molenaar WM, et al. Clinicopathologic assessment of postradiation sarcomas: KIT as a potential treatment. Clin Cancer Res 2003; 9: 2926-32.
23. Yang J-L, Hannan MT, Russell PJ, Crowe PJ. Expression of HER1/EGFR protein in human soft tissue sarcomas. Eur J Surg Oncol 2006; 32: 466-468.
24. Syed SP, Martin AM, Haupt HM, et al. Angiostatin receptor annexin II in vascular tumors including angiosarcoma. Human Pathol 2007; 38: 508-13.

25. Fujimoto M, Kiyosawa T, Murata S, et al. Vascular endothelial growth factor in angiosarcoma. Anticancer Res 1998;18:3725-9.
26. Zietz C, Rossle M, Haas C, et al. MDM-2 oncoprotein overexpression, p53 gene mutation, and VEGF up-regulation in angiosarcomas. Am J Pathol 1998;153:1425-33.
27. Arbiser JL, Larsson H, Claesson-Welsh L, et al. Overexpression of VEGF 121 in immortalized endothelial cells causes conversion to slowly growing angiosarcoma and high level expression of the VEGF receptors VEGFR-1 and VEGFR-2 in vivo. Am J Pathol 2000; 156:1469-76.
28. Thurston G, Gale NW. VEGF and other signalling pathways in developmental and pathologic angiogenesis. Int J Haematol 2004; 80: 7-20.
29. Ferrara N, Kerbel RS. Angiogenesis as a therapeutic target. Nature 2005;438: 967-974.
30. Rafii S. Circulating endothelial precursors: mystery, reality and promise. J Clin Invest 2000; 105: 17.
31. Mancuso P, Burlini A, Pruneri G et al. Resting and activated endothelial cells are increased in the peripheral blood of cancer patients. Blood 2001; 97: 3658-3661.
32. Ribatti D. The involvement of endothelial progenitor cells in tumour angiogenesis. J Cell Mol Med 2004; 8: 294-300.
33. Hong YK, K Foreman K, Shin JW et al. Lymphatic reprogramming of blood vascular endothelium by Kaposi sarcoma-associated herpesvirus. Nat Genet 2004; 36:683-5.
34. Wang HW, et al. Kaposi sarcoma herpesvirus-induced cellular reprogramming contributes to the lymphatic endothelial gene expression in Kaposi sarcoma. Nat Genet 2004; 36: 687-93.
35. Crew AJ, Clark J, Fisher C et al. Fusion of SYT to two genes, SSX1 and SSX2, encoding proteins with homology to the Kruppel-associated box in human synovial sarcoma. EMBO J 1995;14:2333–2340.
36. Nielsen TO, West RB, Linn SC, et al. Molecular characterisation of soft tissue tumours: a gene expression study. Lancet. 2002; 359: 1301-7.
37. Kanjeekal S, Chambers A, Fung MFK, et al, Systemic therapy for advanced uterine sarcoma: A systematic review of the literature. Gynecologic Oncology 2005; 97: 624-637.
38. Hartman JT, Patel S. Recent Developments in Salvage Chemotherapy for Patients with Metastatic Soft Tissue Sarcoma. Drugs 2005; 65: 167-178.
39. Wright JD, Powell MA, Rader JS, et al. Bevacizumab therapy in patients with recurrent uterine neoplasms. Anticancer Res 2007; 27: 3525-8.
40. Inai T, Mancusco M, Hashizume H, et al. Inhibition of vascular endothelial growth factor (VEGF) signalling in cancer causes loss of endothelial fenestrations, regression of tumor vessels, and appearance of basement membrane ghosts. Am J Pathol 2004; 165: 35-52.
41. Rugo HS, Herbst RS, Liu G, et al. Phase I trial of the oral antiangiogenesis agent AG-013736 in patients with advanced solid tumors: pharmacokinetics and clinical results. J Clin Oncol 2005; 23:5474-83.
42. Rixe O, Bukowski RM, Michaelson MD, et al. Axitinib treatment in patients with cytokine-refractory metastatic renal-cell cancer: a phase II study. *Lancet Oncol* 2007; 8: 975–84
43. Cohen EE, Rosen LS, Vokes EE, Kies MS, et al. Axitinib is an active treatment for all histologic subtypes of advanced thyroid cancer: results from a phase II study. J Clin Oncol. 2008, 26(29):4708-13.Epub 2008 Jun 9.
44. Spano JP, Chodkiewicz C, Maurel J et al. Efficacy of gemcitabine plus axitinib compared with gemcitabine alone in patients with advanced pancreatic cancer: an open-label randomised phase II study. *Lancet* 2008; 371:2101-8.
45. Giles FG, Bellamy WT, Estrov Z et al. The anti-angiogenesis agent, AG-013736, has minimal activity in elderly patients with poor prognosis acute myeloid leukemia (AML) or myelodysplastic syndrome (MDS). *Leuk Res* 2006; 30: 801-11.

Appendix 2: NCI Common Terminology Criteria for Adverse Events

Adverse Events will be recorded according to the National Cancer Institute Common Terminology Criteria for Adverse Events version 4.0 (NCI CTCAE, v4.0).

The full NCI CTCAE (v4.0) document is supplied in the Investigator Site Folder and can also be requested from the Axi-STS Trial Office.

It is also available on the National Cancer Institute (NCI) website, at the following address: http://ctep.cancer.gov/reporting/ctc.html.

Appendix 3: Summary of RECIST

a. Measurability of tumour lesions at baseline

Measurable

Tumour lesions: Must be accurately measured in at least one dimension (longest diameter in the plane of measurement is to be recorded) with a minimum size of:

- 10 mm by CT scan (CT scan slice thickness no greater than 5mm).
- 10 mm caliper measurement by clinical exam (lesions which cannot be accurately measured with calipers should be recorded as non –measurable).
- 20mm by chest X –ray.

All measurements should be recorded in metric notation using calipers if clinically assessed. All baseline evaluations should be performed as close as possible to the treatment start and never more than 4 weeks before the beginning of the treatment.

Method of assessment

For the case of skin lesions, documentation by colour photography including a ruler to estimate the size of the lesion is suggested.

b. Tumour response evaluation

Baseline documentation of “Target” and “Non-Target” lesions

When more than one measurable lesion is present at baseline all lesions up to a maximum of five lesions in total (and a maximum of two lesions per organ) representative of all involved organs, should be identified as target lesions and will be recorded and measured at baseline (this means in instances where patients have only one or two organ sites involved a maximum of two and four lesions respectively will be recorded).

Target lesions should be selected on the basis of their size (lesions with the longest diameter), be representative of all involved organs, but in addition should be those that lend themselves to reproducible repeated measurements.

A sum of the diameters (longest for non-nodal lesions, short axis for nodal lesions) for all target lesions will be calculated and reported as the baseline sum diameters. If lymph nodes are to be included in the sum, then as noted above, only the short axis is included into the sum. The baseline sum diameters will be used as reference to further characterise any objective tumour regression in the measurable dimension of the disease.

All other lesions (or sites of disease) including pathological lymph nodes should be identified as non-target lesions and should also be recorded at baseline. Measurements are not required and these lesions should be followed as ‘present’, ‘absent’ or in rare cases ‘unequivocal progression’. In addition, it is possible to record multiple non-target lesions involving the same organ as a single item on the case record form (e.g. ‘multiple enlarged pelvic lymph nodes’ or ‘multiple liver metastases’). but the presence or absence of each should be noted throughout follow-up.

Evaluation of target lesions

Complete Response (CR):	Disappearance of all target lesions. Any pathological lymph nodes (whether target or not-target) must have reduction in short axis to <10mm.
Partial Response (PR):	At least a 30% decrease in the sum of diameters of target lesions, taking as reference the baseline sum diameters...
Progressive disease (PD):	At least a 20% increase in the sum of diameters of target lesions, taking as reference the smallest sum on study (this includes the baseline sum if that is the smallest on study). In addition to the relative increase of 20%, the sum must almost demonstrate an absolute increase of at least 5mm (*Note*: the appearance of one or more new lesions is also considered progression).
Stable Disease (SD):	Neither sufficient shrinkage to qualify for PR nor sufficient increase to qualify for PD taking as references the smallest sum diameters while on study.

Evaluation of non-target lesions

Complete Response (CR):	Disappearance of all non-target lesions and normalisation of tumour marker level. All lymph nodes must be non-pathological in size (<10 mm short axis).
Non-CR/Non-PD	Persistence of one or more non-target lesion(s) or/and maintenance of tumour marker level above the normal limits.
Progressive disease (PD):	Unequivocal progression of existing non-target lesions. (*Note*: the appearance of one or more new lesions is also considered progression).

Evaluation of best overall response

The best overall response is the best response recorded from the start of the study treatment until the end of treatment taking into account any requirement for confirmation. The patient's best overall response assignment will depend on the findings of both target and non-target disease and will also take into consideration the appearance.

Time point response: patients with target (+/- non target) disease

Target lesions	Non-Target lesions	New Lesions	Overall response
CR	CR	No	CR
CR	Non-CR/non-PD	No	PR
CR	Not evaluated	No	PR
PR	Non-PD or not all evaluated	No	PR
SD	Non-PD or not all evaluated.	No	SD
Not all evaluated	Non-PD	No	NE
PD	Any	Yes or No	PD
Any	PD	Yes or No	PD
Any	Any	Yes	PD
CR= complete response, PR= partial response, SD= stable disease, PD= progressive disease, NE= inevaluable.			

Time point response: patients with non-target disease only

Non-Target lesions	New Lesions	Overall response
CR	No	CR
Non-CR/non-PD	No	Non-CR/non-PD [a]
Not all evaluated	No	NE
Unequivocal PD	Yes or No	PD
Any	Yes	PD
CR= complete response, PD= progressive disease, NE= inevaluable<br>a 'Non-CR/non PD' is preferred over 'stable disease' for non-target disease since SD is increasingly used as endpoint for assessment of efficacy in some trials so to assign this category when no lesions can be measured is not advised.		

Appendix 4: WHO Performance Status Scale

Description	Grade
Fully active, able to carry on all pre-disease activities without restriction.	**0**
Restricted in physically strenuous activity but ambulatory and able to carry out work of a light or sedentary nature e.g. light housework, office work	**1**
Ambulatory and capable of all self-care but unable to carry out any work activities. Up and about more than 50% of waking hours.	**2**
Capable of only limited self care, confined to bed or chair more than 50% of waking hours.	**3**
Completely disabled. Cannot carry on any self-care. Totally confined to bed or chair.	**4**

Appendix 5: Cockcroft-Gault Formula

Males : CrCl (mL/min) = $\dfrac{[(140 - age) \times weight]}{SeCr} \times 1.23$

Females : CrCl (mL/min) = $\dfrac{[(140 - age) \times weight]}{SeCr} \times 1.04$

Where age is in years, weight is in kg and SeCr is in μmol/l.

CrCl= Creatinine Clearance
SeCr= Serum Creatinine

Appendix 6: Axitinib Clinical Interactions

Inhibitors and Inducers of CYP3A

Inhibitors		Inducers
Amiodarone	Indinavir	Barbiturates
Cimetidine	Itraconazole	Carbamazepine
Ciprofloxacin	Ketoconazole	Dexamethasone
Clarithromycin	Miconazole	Efavirenz
Delaviridine	Voriconazole	Glucocorticoids
Diltiazem	Mifepristone	Modafinil
Erythromycin	Nefazodone	Nevirapine
Fluconazole	Nelfinavir	Phenobarbital
Fluvoxamine	Norfloxacin	Phenytoin
Gestodene	Ritonavir	Rifampicin
Grapefruit juice	Saquinavir	St. John’s Wort
Mibefradil	Lopinavir	Felbamate
Troleandomycin	Verapamil	Omeprazole
Telithromycin		Primidone
		Rifabutin
		Troglitazone

Inducers of CYP1A2

Barbiturates
Carbamazepine
Modafinil
Nafcillin
Phenobarbital
St. John’s Wort
Omeprazole
Primidone
Rifampicin
Rifabutin

Supplementary appendix 2

Axitinib treatment details split by stratum.

	Angiosarcoma N=39 (%)	Leiomyo-sarcoma N=36 (%)	Synovial Sarcoma N=36 (%)	Other Sarcoma N=34 (%)
Number of patients starting trial treatment	38	35	34	31
Time to treatment (days)				
Mean (s.d.)	2.4 (2.9)	3.7 (6.6)	2.9 (5.0)	3.8 (3.9)
Median	1.0	1.0	1.0	3.0
Range	0.0, 9.0	0.0, 34.0	0.0, 25.0	0.0, 18.0
Cycles received				
Mean (s.d.)	7.7 (10.5)	4.9 (3.7)	6.4 (8.2)	4.8 (5.1)
Median	3.0	3.0	3.0	3.0
Range	1.0, 53.0	1.0, 15.0	1.0, 34.0	1.0, 24.0
Dose reductions				
Number	20	17	9	12
Number of patients	17	14	7	11
Treatment interruptions (cycle 1)				
Number	22	18	16	23
Number of patients	18	11	11	15
Treatment interruptions (cycles 2-6)				
Number	33	22	13	15
Number of patients	19	15	9	12
Treatment interruptions (after cycle 6)				
Number	29	16	23	10
Number of patients	7	6	5	4
Patients who discontinued treatment	37 (97)	35 (100)	34 (100)	31 (100)
Reasons for discontinuation				
Disease progression	22 (59)	25 (71)	25 (74)	23 (74)
Toxicity	5 (14)	5 (14)	4 (12)	2 (6)
Death	0	2 (6)	1 (3)	0
Dose delay of >14 days	2 (5)	1 (3)	0	1 (3)
Patient withdrew consent	1 (3)	1 (3)	0	0
Non-disease related illness	1 (3)	0	0	3 (10)
Disease progression and death	0	0	1 (3)	0
Disease progression and non-disease related illness	1 (3)	0	0	0
Disease progression and toxicity	0		1 (3)	0
Toxicity and further treatment not in patients' best interest	1 (3)	0	0	0
Toxicity, dose delay >14 days and further treatment not in their best interest patient	0	1 (3)	0	0
Non-disease related illness and death	1 (3)	0	0	0
Further treatment not in patients' best interests	0	0	1 (3)	0
Compliance <50% over a 4-week period,	0	0	1 (3)	0
Patient refusal	2 (5)	0	0	0
Reason not provided	1 (3)	0	1 (3)	2 (5)
Patients who received further anti-tumour treatments	19 (51)	17 (49)	19 (56)	18 (58)

s.d., standard deviation

Supplementary appendix 3

The presence of non-target lesions at baseline were collected, then at each disease evaluation visit the presence, number, and location of any new lesions were reported.

Table S3A: New lesions identified in patients

Sarcoma	New Lesion Location	Details	N	Patients Affected
Angiosarcoma	Liver		5	5
	Skin		4	4
	Lymph		3	3
	Breast		2	1
	Lung		1	1
	Bone		1	1
	Brain		1	1
	Pelvic girdle		1	1
	Other intra-abdominal	Left nephrectomy bed	1	1
	Other intra-abdominal	Peritoneal quadrant	1	1
	Other intra-abdominal	Soft tissue on cortical surface of left kidney	1	1
	Other trunk	Vertebral canal/sacral neural foramen	1	1
Leiomyosarcoma	Lung		3	3
	Liver		2	2
	Head & Neck		2	1
	Liver	Portahepatitis	1	1
	Bone		1	1
	Other soft tissue	Subcutaneous nodules	1	1
	Pelvic girdle		1	1
	Other intra-abdominal		1	1
	Other intra-abdominal	Left paracolic gutter	1	1
	Other intra-abdominal	Left upper quadrant deposit abdomen	1	1
	Other intra-abdominal	Perirectal mass	1	1
	Other intra-abdominal	Peritoneum	1	1
	Other intra-abdominal	Right adrenal	1	1
	Other intra-abdominal	Spleen	1	1
	Other	Mediastinum	1	1
Synovial Sarcoma	Lung		5	5
	Lymph		2	2
	Other intra-abdominal		2	1
	Other soft tissue	Right acetabulum	1	1
	Pelvic girdle		1	1
Other Sarcoma	Liver		2	2
	Lung		2	2
	Pelvic girdle		2	1
	Lymph		1	1
	Bone		1	1
	Brain		1	1
	Other trunk	Chest wall	1	1

Supplementary appendix 4

Patients were eligible for the Axi-STS trial if, at baseline, a WHO performance score of 0, 1 or 2 was observed. Following this, patients' performance status was measured weekly during cycle 1 of treatment and additionally at the start of each four-week treatment cycle up to the cycle 6.

Table S4A: Change in performance status score from baseline to cycle 3

	WHO Performance Status at Baseline			Missing	Total
Change, n (%)	0	1	2		
**Angiosarcoma**					
Improvement (1 point)	0 (0.0)	2 (12.5)	0 (0.0)	0 (0.0)	2 (5.6)
No change	6 (35.3)	6 (37.5)	0 (0.0)	0 (0.0)	12 (33.3)
Worsened (1 point)	4 (23.5)	2 (12.5)	0 (0.0)	0 (0.0)	6 (16.7)
Worsened (2 points)	1 (5.9)	0 (0.0)	0 (0.0)	0 (0.0)	1 (2.8)
Not known	6 (35.3)	6 (37.5)	3 (100.0)	0 (0.0)	15 (41.7)
Total*	17	16	3	0	36
**Leiomyosarcoma**					
Improvement (1 point)	0 (0.0)	3 (14.3)	0 (0.0)	0 (0.0)	3 (8.6)
No change	6 (60.0)	12 (57.1)	1 (33.3)	0 (0.0)	19 (54.3)
Worsened (1 point)	3 (30.0)	1 (4.8)	0 (0.0)	0 (0.0)	4 (11.4)
Worsened (2 points)	0 (0.0)	0 (0.0)	0 (0.0)	0 (0.0)	0 (0.0)
Not known	1 (10.0)	5 (23.8)	2 (66.7)	1 (100.0)	9 (25.7)
Total*	10	21	3	1	35
**Synovial sarcoma**					
Improvement (1 point)	0 (0.0)	0 (0.0)	0 (0.0)	0 (0.0)	0 (0.0)
No change	7 (47.7)	10 (58.8)	1 (50.0)	0 (0.0)	18 (52.9)
Worsened (1 point)	4 (26.7)	1 (5.9)	0 (0.0)	0 (0.0)	5 (14.7)
Worsened (2 points)	0 (0.0)	0 (0.0)	0 (0.0)	0 (0.0)	0 (0.0)
Not known	4 (26.7)	6 (35.3)	1 (50.0)	0 (0.0)	11 (32.4)
Total*	15	17	2	0	34
**Other sarcoma**					
Improvement (1 point)	0 (0.0)	2 (8.3)	0 (0.0)	0 (0.0)	2 (6.5)
No change	4 (66.7)	10 (41.7)	0 (0.0)	0 (0.0)	14 (45.2)
Worsened (1 point)	1 (16.7)	3 (12.5)	0 (0.0)	0 (0.0)	4 (12.9)
Worsened (2 points)	0 (0.0)	0 (0.0)	0 (0.0)	0 (0.0)	0 (0.0)
Not known	1 (16.7)	9 (37.5)	1 (100.0)	0 (0.0)	11 (35.5)
Total*	6	24	1	0	31
**All Sarcomas**					
Improvement (1 point)	0 (0.0)	7 (9.0)	0 (0.0)	0 (0.0)	7 (5.1)
No change	23 (47.9)	38 (48.7)	2 (22.2)	0 (0.0)	63 (46.3)
Worsened (1 point)	12 (25.0)	7 (9.0)	0 (0.0)	0 (0.0)	19 (14.0)
Worsened (2 points)	1 (2.1)	0 (0.0)	0 (0.0)	0 (0.0)	1 (0.7)
Not known	12 (25.0)	26 (33.3)	7 (77.8)	1 (100.0)	46 (33.8)
Total*	48	78	9	1	136

* Total number of patients included are those within the safety analysis population

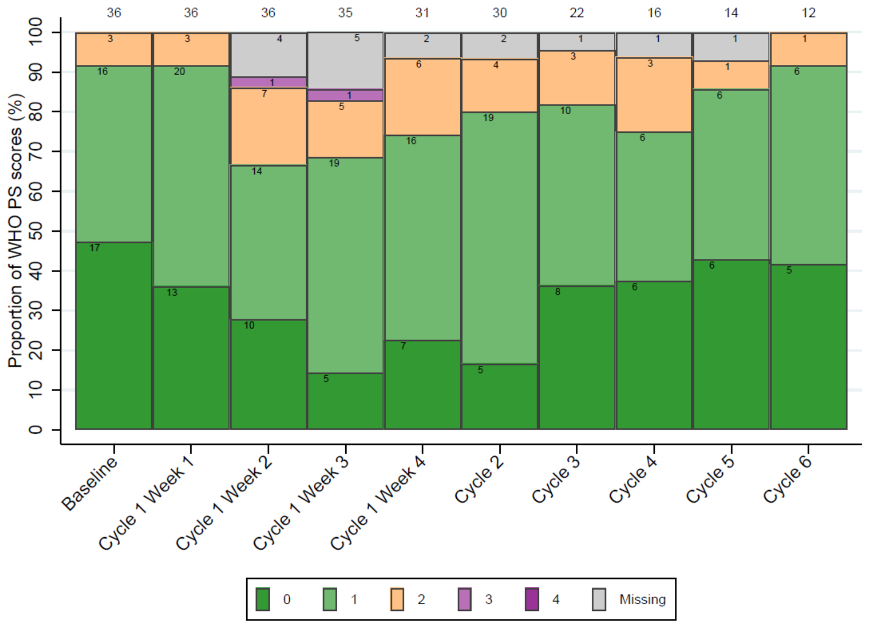

Figure S4A: Percentage of patients' performance status from baseline to the end of cycle 6 of treatment within angiosarcoma patients

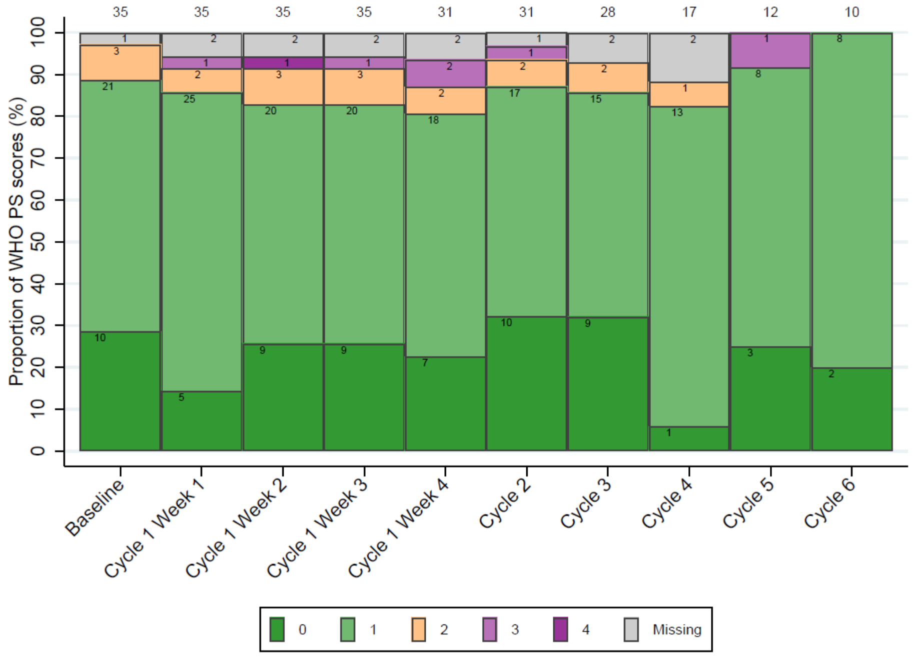

Figure S4B: Proportion of patients' performance status from baseline to the end of cycle 6 of treatment within leiomyosarcoma patients

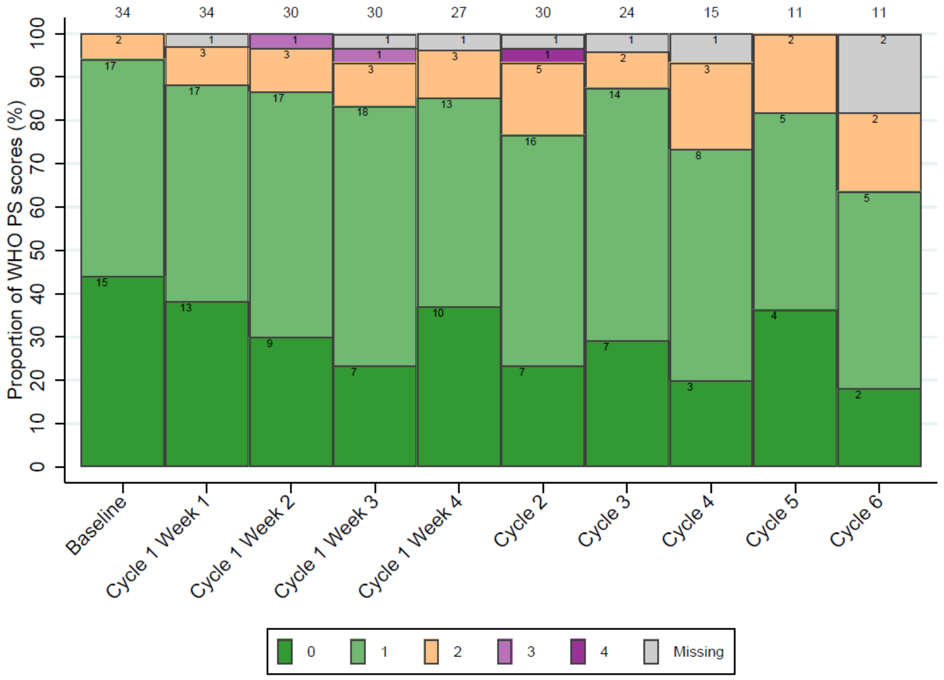

Figure S4C: Proportion of patients' performance status from baseline to the end of cycle 6 of treatment within synovial sarcoma patients

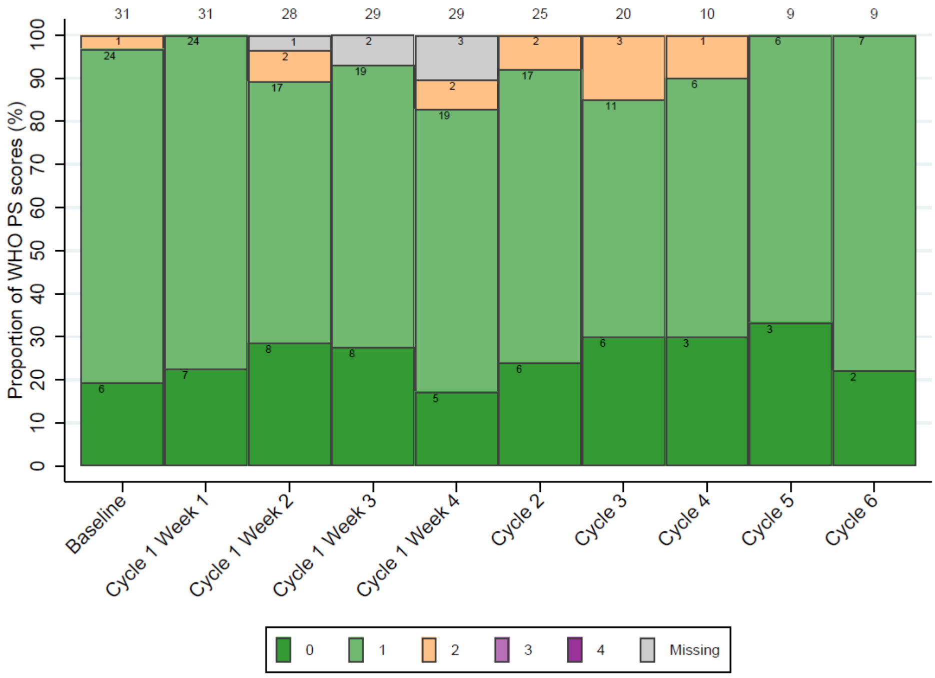

Figure S4D: Proportion of patients' performance status from baseline to the end of cycle 6 of treatment within patients with other sarcoma subtypes

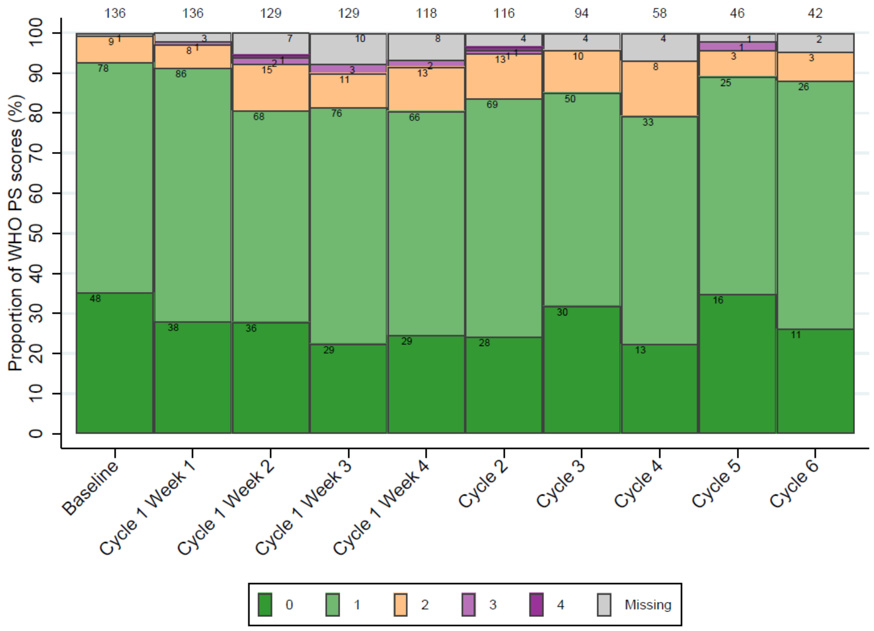

Figure S4E: Proportion of patients' performance status from baseline to the end of cycle 6 of treatment within all registered sarcoma patients

Supplementary appendix 5

Toxicity profiles recorded during the Axi-STS trial.

Table S5A: Grade $\geq$3 and unknown toxicities

Stratum	CTCAE Category	Event	Grade	Number of Events	Number Affected
Angiosarcoma	Blood and lymphatic system disorders	Anemia	3	1	1
	Ear and labyrinth disorders	Hearing impaired	Not known	1	1
	Gastrointestinal disorders	Diarrhea	3	1	1
		Dry mouth	Not known	1	1
		Mucositis oral	3	1	1
		Nausea	Not known	1	1
		Vomiting	Not known	1	1
	General disorders and administration site conditions	Fatigue	3	9	7
		Fatigue	Not known	1	1
		Gait disturbance	3	1	1
		Pain	3	2	1
		Pain	Not known	1	1
	Injury, poisoning and procedural complications	Wound complication	3	4	1
	Investigations	Alanine aminotransferase increased	3	1	1
		GGT increased	3	1	1
		Weight loss	3	4	2
	Metabolism and nutrition disorders	Anorexia	3	3	2
		Anorexia	Not known	1	1
		Hyponatremia	3	1	1
	Musculoskeletal and connective tissue disorders	Arthralgia	3	1	1
		Arthralgia	Not known	1	1
		Back pain	3	1	1
		Bone pain	3	1	1
		Neck pain	3	1	1
		Pain in extremity	3	1	1
		Trismus	Not known	1	1
	Nervous system disorders	Dysgeusia	Not known	1	1
		Paresthesia	Not known	1	1
	Respiratory, thoracic and mediastinal disorders	Cough	Not known	1	1
		Dyspnea	3	3	2
		Dyspnea	Not known	1	1
		Haemoptysis	Not known	1	1
		Pleural effusion	3	1	1
		Pleuritic pain	3	2	1
		Voice alteration	3	1	1
	Skin and subcutaneous tissue disorders	Bullous dermatitis	3	1	1
	Vascular disorders	Hypertension	3	18	10
Leiomyosarcoma	Eye disorders	Blurred vision	Not known	2	1
	General disorders and administration site conditions	Fatigue	3	10	6
		Fever	3	1	1
		Pain	3	2	1
		Pain	Not known	1	1
	Gastrointestinal disorders	Diarrhea	3	2	2

Stratum	CTCAE Category	Event	Grade	Number of Events	Number Affected
	Infections and infestations	Upper respiratory infection	3	1	1
	Investigations	Weight loss	3	5	3
	Metabolism and nutrition disorders	Anorexia	3	4	2
		Hypercalcemia	3	1	1
		Hypercalcemia	4	1	1
		Hyperglycemia	3	1	1
		Hypophosphatemia	3	1	1
	Musculoskeletal and connective tissue disorders	Arthralgia	3	6	2
		Myalgia	3	1	1
	Nervous system disorders	Headache	3	2	2
	Psychiatric disorders	Depression	3	1	1
		Mood alteration	3	1	1
	Respiratory, thoracic and mediastinal disorders	Dyspnea	3	1	1
		Sore throat	3	1	1
	Vascular disorders	Hypertension	3	14	6
Synovial sarcoma	Eye disorders	Retinal vascular disorder	3	1	1
	Gastrointestinal disorders	Abdominal pain	Not known	1	1
		Diarrhea	3	1	1
		Lower gastrointestinal hemorrhage	3	1	1
	General disorders and administration site conditions	Fatigue	3	4	2
		Fever	3	2	1
		Non-cardiac chest pain	3	1	1
		Pain	3	2	1
	Infections and infestations	Lung infection	4	1	1
	Investigations	GGT increased	3	2	2
	Metabolism and nutrition disorders	Hypocalcemia	3	1	1
	Musculoskeletal and connective tissue disorders	Pain in extremity	3	1	1
	Nervous system disorders	Peripheral motor neuropathy	3	1	1
		Peripheral sensory neuropathy	3	1	1
		Peripheral sensory neuropathy	Not known	1	1
		Spinal cord compression	3	1	1
	Respiratory, thoracic and mediastinal disorders	Bronchopulmonary hemorrhage	3	1	1
		Cough	Not known	1	1
		Dyspnea	3	3	2
		Dyspnea	4	1	1
		Pleural effusion	3	1	1
		Pneumothorax	3	2	2
	Vascular disorders	Hypertension	3	2	1
Other sarcoma	Blood and lymphatic system disorders	Anemia	3	1	1
	Gastrointestinal disorders	Abdominal pain	3	3	2
		Colonic fistula	3	2	1
		Diarrhea	3	3	3
		Mucositis oral	3	2	2
		Vomiting	3	1	1
	General disorders and administration site conditions	Fatigue	3	10	6
		Pain	Not known	1	1
	Musculoskeletal and connective tissue disorders	Back pain	3	1	1

Stratum	CTCAE Category	Event	Grade	Number of Events	Number Affected
	Respiratory, thoracic and mediastinal disorders	Pleural hemorrhage	3	1	1
		Pneumothorax	3	2	1
	Vascular disorders	Hypertension	3	14	6
		Leaking intra-tumoural renal artery	3	1	1
		Thromboembolic event	3	2	2

Table S5B: Adverse events experienced in at least 10% of patients across all four strata

Toxicity	Grade 1	Grade 2	Grade 3	Grade 4	Missing	Total N (%*)
Fatigue	40	53	21	0	0	114 (84%)
Hypertension	22	49	23	0	0	94 (69%)
Nausea	53	19	0	0	0	72 (53%)
Dyspnea	44	19	5	1	0	69 (50.74%)
Anorexia	41	23	4	0	0	68 (50%)
Mucositis oral	45	20	3	0	0	68 (50%)
Diarrhea	38	17	7	0	0	62 (46%)
Cough	47	9	0	0	1	57 (42%)
Voice alteration	48	7	1	0	0	56 (41%)
Pain	27	23	3	0	1	54 (40%)
Constipation	38	14	0	0	0	52 (38%)
Headache	40	7	2	0	0	49 (36%)
Weight loss	30	13	5	0	0	48 (35%)
Arthralgia	29	9	3	0	0	41 (30%)
Proteinuria	26	12	0	0	0	38 (28%)
Abdominal pain	21	13	2	0	0	36 (26%)
Vomiting	24	10	1	0	0	35 (26%)
Palmar-plantar erythrodysesthesia syndrome	21	12	0	0	0	33 (24%)
Pain in extremity	13	6	2	0	0	21 (15%)
Non-cardiac chest pain	11	8	1	0	0	20 (15%)
Urinary tract infection	5	11	0	0	0	16 (12%)
Dyspepsia	14	1	0	0	0	15 (11%)
Back pain	10	2	2	0	0	14 (10.29%)
Dry mouth	12	1	0	0	1	14 (10%)
Dry skin	12	2	0	0	0	14 (10%)

* Percentage calculated as number of patients affected over the total number of patients included within the safety analysis population (136)

Supplementary appendix 6

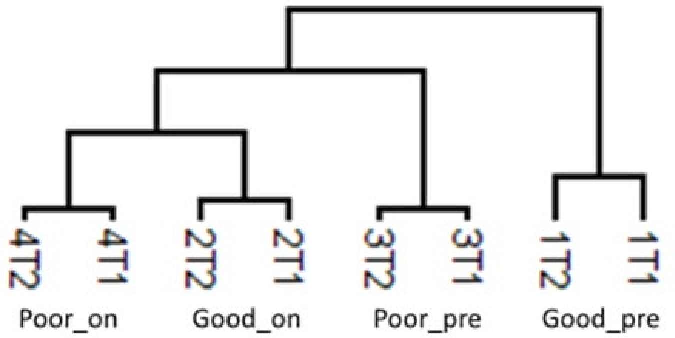

Figure S6A: Hierarchical cluster analysis of the 4 sample groups studied

Samples were clustered based on the similarity of their protein expression profiles observed in log10 of the iTRAQ ratios, and a dendrogram generated to indicate the relationship between the samples. Each sample consisted of pooled serum from 4 leiomyosarcoma patients retrospectively classified as good responders (PFS on axitinib ≥4 months) and poor responders (PFS on axitinib <4 months), and collected pre-treatment and at 4 weeks on treatment; replicate samples were analysed. In the dendrogram, Poor_on shows poor responders on treatment, Good_on shows good responders on treatment, Poor_pre shows poor responders pre-treatment, and Good_pre shows good responders pre-treatment. This figure shows the good responders pre-treatment clustering away from the other samples.

Table S6A: Significant fold change in protein expression between good and poor responders pre-treatment

Increased in Poor Pre samples relative to Good Pre samples, p value 0.05								
T1, T2 technical replicates			1T1	1T2	3T1	3T2		
**Uniprot**	**#unique peptides**	**#peptides for quant**	**Good_Pre_T1**	**Good_Pre_T2**	**Poor_Pre_T1**	**Poor_Pre_T2**	**Protein names**	**Gene names**
P02751	36	6	1.00	1.25	3.81	3.48	Fibronectin	FN1
P63261	14	5	1.00	0.84	2.74	2.56	Actin, cytoplasmic 2	ACTG1
P20742	34	17	1.00	1.01	2.17	1.88	Pregnancy zone protein	PZP
P00738	26	24	1.00	0.98	1.86	1.73	Haptoglobin	HP
P00748	9	5	1.00	0.95	1.76	1.70	Coagulation factor XII	F12
P02671	27	24	1.00	0.94	1.70	1.64	Fibrinogen alpha chain	FGA
P05546	27	25	1.00	1.09	1.57	1.54	Heparin cofactor 2	SERPIND1
P68871	11	9	1.00	1.10	1.53	1.27	Hemoglobin subunit beta	HBB
D6RF35	31	30	1.00	1.04	1.47	1.40	Vitamin D-binding protein	GC
P01024	166	151	1.00	1.05	1.40	1.37	Complement C3	C3
P04114	279	212	1.00	1.01	1.38	1.33	Apolipoprotein B-100	APOB
A0A096LPE2	5	3	1.00	1.01	1.37	1.17	Protein SAA2-SAA4	SAA2-SAA4
P80108	20	11	1.00	0.92	1.37	1.35	Phosphatidylinositol-glycan-specific phospholipase D	GPLD1
P02748	20	16	1.00	1.00	1.35	1.25	Complement component C9	C9
H0YAC1	19	13	1.00	1.07	1.34	1.45	Plasma kallikrein	KLKB1
P08603	51	35	1.00	1.06	1.30	1.26	Complement factor H	CFH
P02763	13	12	1.00	0.99	1.24	1.25	Alpha-1-acid glycoprotein 1	ORM1
P01031	84	54	1.00	1.00	1.19	1.25	Complement C5	C5
Decreased in Poor Pre samples relative to Good Pre samples, p value 0.05								
P02647	40	37	1.00	1.03	0.85	0.83	Apolipoprotein A-I	APOA1
P02750	14	14	1.00	1.03	0.78	0.81	Leucine-rich alpha-2-glycoprotein	LRG1
B4E1Z4	61	59	1.00	0.96	0.74	0.72	Uncharacterized protein, highly similar to Complement factor B	
A0A087WYJ9	16	13	1.00	0.96	0.69	0.65	Ig mu chain C region	IGHM
P06727	44	42	1.00	0.92	0.67	0.60	Apolipoprotein A-IV	APOA4
P10643	30	25	1.00	1.04	0.66	0.70	Complement component C7	C7
Q06033	30	24	1.00	0.90	0.63	0.66	Inter-alpha-trypsin inhibitor heavy chain H3	ITIH3
P01834	7	7	1.00	1.00	0.38	0.38	Ig kappa chain C region	IGKC
P01876	10	9	1.00	1.06	0.35	0.31	Ig alpha-1 chain C region	IGHA1

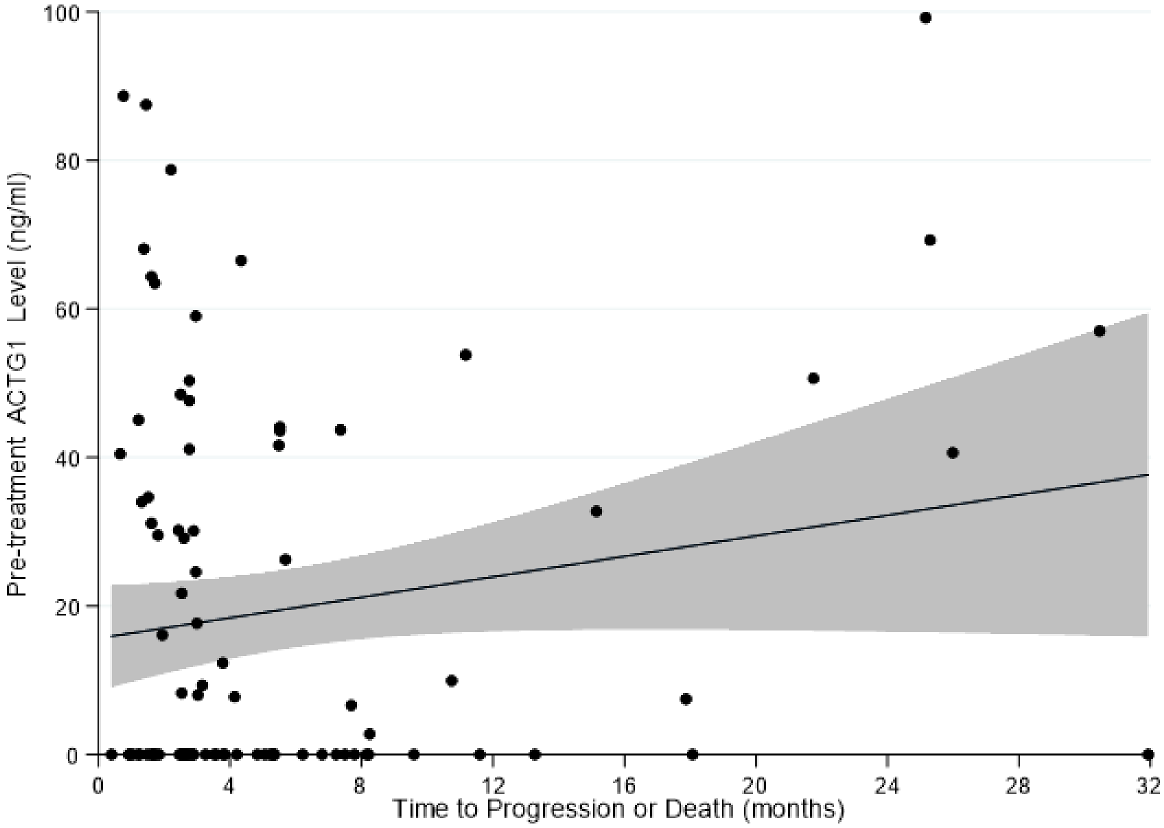

Figure S6B: Expression levels of circulating ACTG1

Scatter plot of pre-treatment ACTG1 concentration (ng/ml) at time of tumour progression or patients' death. No patients were censored. All study patients who had their pre-treatment ACTG1 concentration determined are plotted (N=93; 46 detectable and 47 undetectable).

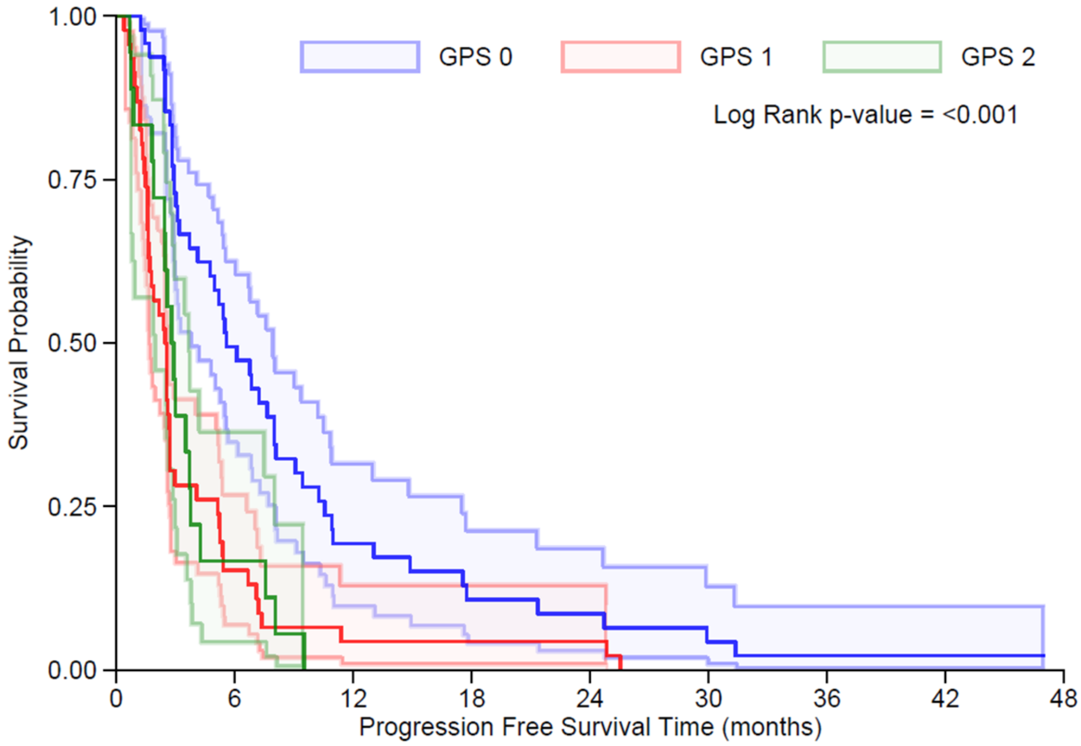

Number at risk

GPS 0	49	(24)	23	(14)	9	(4)	5	(1)	4	(2)	2	(1)	1	(0)	1	(0)	0
GPS 1	46	(39)	7	(5)	2	(0)	2	(0)	2	(2)	0	(0)	0	(0)	0	(0)	0
GPS 2	18	(15)	3	(3)	0	(0)	0	(0)	0	(0)	0	(0)	0	(0)	0	(0)	0

Figure S6C: Progression-free survival by modified Glasgow Prognostic Score

Kaplan–Meier analysis of progression-free survival defined as the time from trial entry to the date when disease progression was first observed including deaths, whatever the cause, split by patients classified as per the modified Glasgow prognostic score (GPS).[19]
